# Impaired hepatic metabolism in Hereditary Fructose Intolerance confers fructose-independent risk for steatosis and hypertriglyceridemia

**DOI:** 10.1016/j.molmet.2025.102310

**Published:** 2025-12-19

**Authors:** Melissa A. Fulham, John D. Griffin, Sylvie Perez, Zhongyuan Sun, Natalie A. Daurio, Gang Xing, Michelle F. Clasquin, Melissa R. Miller, Craig L. Hyde, Scott P. Kelly, Magalie Boucher, Rachel Poskanzer, Ramya Gamini, Evanthia Pashos, Ying Zhang, Elaine Kuang, Josh Fienman, Kendra K. Bence, Gregory J. Tesz

**Affiliations:** 1 Internal Medicine Research Unit, Pfizer Inc., Cambridge, MA, USA; 2 Early Clinical Development, Pfizer Inc., Groton, CT, USA; 3 Global Medical Epidemiology, Pfizer Inc., New York, NY, USA; 4 Drug Safety Research and Development, Pfizer Inc., Cambridge, MA, USA; 5 Inflammation and Remodeling, Pfizer Inc., Cambridge, MA, USA

**Keywords:** Hereditary fructose intolerance, Aldolase B, Fructose, Steatosis, Hyperlipidemia

## Abstract

**Objectives:**

Hereditary fructose intolerance (HFI), caused by Aldolase B deficiency, is a rare genetic disorder where fructose exposure leads to severe metabolic pathologies including Type-2 diabetes and liver steatosis. Despite adhering to fructose-free diets, some individuals still present with disease. Using a rat model of HFI we demonstrate that fructose independent pathologies exist and identify the molecular pathways driving disease.

**Methods:**

*Aldob* was deleted in Sprague Dawley rats using CRIPSR/Cas9 (AldoB-KO). Phenotypic, metabolomic and transcriptomic studies were conducted to identify mechanisms promoting fructose-independent pathologies. Potential molecular causes were tested using pharmacologic inhibitors and ASOs.

**Results:**

Deletion of *Aldob* caused hepatic steatosis, fibrosis and stunted growth in rats weaned on low fructose chow recapitulating human HFI. On fructose-free chow, AldoB-KO rats were phenotypically normal. However, upon fasting, male and female AldoB-KO rats developed hepatic steatosis and hyperlipidemia due to impaired fatty acid oxidation (FAOx) and elevated de novo lipogenesis (DNL). Transcriptional and metabolomic profiling revealed increased hepatic Carbohydrate Response Element Binding Protein (ChREBP) activation in AldoB-KO rats due to glycolytic metabolite accumulation caused by impaired gluconeogenesis. Treatment with Acetyl-CoA Carboxylase (ACC) and Diacylglycerol Acyl Transferase 2 (DGAT2) inhibitors reduced hepatic lipids and plasma triglycerides in AldoB-KO rats. Finally, using electronic health records we observed increased metabolic dysfunction-associated steatohepatitis (MASH) diagnosis in individuals with HFI.

**Conclusions:**

*Aldob* deletion caused fructose-independent hyperlipidemia and steatosis upon fasting in rats. Individuals with HFI may have risk for hepatic disease and hyperlipidemia even upon fructose abstinence suggesting additional therapies may be needed to mitigate disease.

## Highlights

• Aldolase B deficiency causes Hereditary Fructose Intolerance (HFI), a disease where fructose exposure causes severe adverse reactions in people.
• Despite fructose abstinence, individuals with HFI are at risk for developing Type-2 diabetes and often have persistent hepatic steatosis and fibrosis.
• In rats lacking *Aldob* maintained on a fructose-free chow, overnight fasting increases plasma and hepatic triglycerides.
• Loss of *Aldob* causes impaired fatty acid oxidation and elevated de novo lipogenesis upon fasting due to a block in hepatic gluconeogenesis and accumulation of metabolites.
• Pharmacologic strategies to suppress de novo lipogenesis and enhance fatty acid oxidation partially reversed plasma and hepatic triglycerides in fasted rats lacking *Aldob.*

## 1 Introduction

The aldolase reaction is a key step in glycolysis, facilitating the conversion of Fructose-1,6 diphosphate (F1,6P) into glyceraldehyde phosphate (GAP) and dihydroxyacetone phosphate (DHAP) [1]. Three mammalian aldolase isoforms, A (ALDOA), B (ALDOB) and C (ALDOC), which vary in tissue expression and catalytic properties, mediate this reaction. While ALDOC is only expressed in the brain and fetal tissues, ALDOA is ubiquitous and ALDOB is restricted to the liver, intestines, and kidney [2,3]. Though the forward reaction occurs in all cells, the reverse condensation of DHAP and GAP to generate F1,6P also occurs and is required to produce glucose in hepatocytes [4]. In addition to hydrolyzing F1,6P, ALDOB also hydrolyzes Fructose-1 phosphate (F1P), the primary metabolite of fructose, making the enzyme essential for fructose metabolism [5].

Deficiency of ALDOB is the molecular cause of Hereditary Fructose Intolerance (HFI), a rare genetic condition present in about 1:20,000 live births [6]. Individuals with HFI suffer from acute hypoglycemia, severe abdominal pain, and diarrhea following fructose exposure [[7], [8], [9]]. Most symptoms disappear with dietary elimination of fructose, yet some pathologies such as hepatic steatosis and cirrhosis persist for years even after complete removal of dietary fructose, as evidenced by reports of hepatic cirrhosis, rachitic bone disease and stunted growth in subjects with HFI [9]. Increased risk for Type-2 diabetes also exists for subjects with HFI despite elimination of dietary fructose [10]. It has been hypothesized that that accidental fructose exposure due to the pervasiveness of fructose in modern diets drives the increased risk for these pathologies [8]. However, it has been anecdotally noted that people with HFI develop a Pavlovian aversion to “sweet” foods and are adept at avoiding sugar exposure [11], questioning whether common hepatic pathologies are alternatively due to inherent defects in hepatic metabolism.

Here we investigate fructose-independent metabolic dysfunction that develops in fasting ALDOB deficient (AldoB-KO) rats, a model of HFI. Fasting AldoB-KO rats revealed defects in hepatic lipid and carbohydrate metabolism that underly the increased risk for metabolic disease observed in people with HFI.

## 2 Materials and methods

### 2.1 Animals

All procedures performed on animals were in accordance with regulations and established guidelines and were reviewed and approved by an Institutional Animal Care and Use Committee or through an ethical review process. AldoB-KO (*Aldob*^*−/−*^) rats were generated at Taconic Biosciences using CRISPR/Cas9 to delete exons 3–5 in the *Aldob* gene and induce a frameshift with a premature stop codon in exon 6. Sprague–Dawley rat zygotes were injected with Cas9 protein, trans-activating CRISPR RNA (tracrRNA), and sgRNAs upstream of exon 3 and downstream of exon 5 in the *Aldob* gene. Embryos were transferred to female Sprague–Dawley rats and deletion of the target region confirmed in live offspring. Potential-off target effects were investigated through sequencing. All rats were maintained in standard housing conditions and fed a fructose-free diet (Research Diets Inc., #D16121902i). For the low-fructose exposure, animals were fed diet 2920X (Envigo Teklad). Studies were conducted in age- and sex-matched WT and AldoB-KO rats. Rats were randomized by weight into groups.

For fasting studies, rats were fasted overnight for 18 h and then euthanized via CO_2_ asphyxiation followed by exsanguination and bilateral pneumothorax alongside *ad libitum* fed controls. Prior to euthanasia, blood was collected via the tail vein into Microvette CB300 K2 EDTA tubes (Sarstedt, #16.444.100) for plasma isolation. Post-mortem cardiac blood was collected in BD Vacutainer K2 EDTA tubes (BD, #367861) for plasma isolation and tissues were excised rapidly and flash frozen in liquid nitrogen. For the fructose tolerance test, rats received an oral bolus of fructose (1 g/kg at 5 mL/kg) and were euthanized at 0 min (no fructose control), 30 min, and 120 min post-bolus by conscious decapitation. Trunk blood was collected in BD Vacutainer K2 EDTA tubes (BD, #368589) for plasma isolation. For metabolite analysis, animals were anesthetized by intraperitoneal injection of pentobarbital and livers were flash frozen *in situ* using pre-cooled clamps in fully anesthetized rats. Rats were then euthanized by exsanguination and bilateral pneumothorax.

### 2.2 Glycerol and pyruvate/lactate tolerance tests

Prior to dosing, glucose was measured on the AlphaTrak2 glucometer (Zoetis) via the tail vein in fasted animals. Animals received an intraperitoneal dose of either 2 g/kg glycerol (Sigma #G5516), 2 g/kg of a 1:10 (mole:mole) pyruvate/lactate (Sigma #P5280 and #71718) solution, or an equivalent volume of saline. Glucose was measured at 15-, 30-, 60-, and 120-minutes post-injection.

### 2.3 Small-molecule treatments

All small-molecule inhibitors were suspended in 0.5% methylcellulose and administered orally at 5 mL/kg. The ACC inhibitor GS-0976 [12] was administered at 30 mg/kg at the start of an overnight fast. A second dose was administered 12 h later, and the rats were sacrificed 16 h post fast initiation the following morning. The KHK inhibitor PF-06835919 was administered at 15 mg/kg BID for one day prior to an overnight fast. Animals were euthanized the following morning.

### 2.4 ChREBP-ASO treatment

The sequences for the ChREBP (CAGGGCTCTAAGCCATGCAC) and control (CCTTCCCTGAAGGTTCCTCC) ASOs have been previously described [13]. ASOs were resuspended in sterile saline. Rats were maintained in a reverse light/dark cycle for this study. Rats were dosed subcutaneously with either the ChREBP ASO (75 mg/kg) or the control ASO (75 mg/kg) twice, three days apart. 30 h post second dose, rats were fasted overnight and euthanized the following morning.

### 2.5 Western blot analysis

Tissue was homogenized in 50 mM Tris–HCl (pH 7.4), 150 mM NaCl, 2 mM EDTA, 1% Triton X-100, and 0.5% cholate supplemented with cOmplete mini EDTA-free protease inhibitor cocktail tablets (Sigma #11836170001) and PhosSTOP tablets (Sigma #PHOSS-RO) using the TissueLyser II (Qiagen, #85300). Lysates were centrifuged at 14,000 RPM for 10 min at 4 °C and supernatants were transferred to new tubes. Protein concentration was determined using the Pierce BCA Protein Assay Kit (Thermo Fisher, #23227). 20 μg of sample was denatured in 2x NuPAGE LDS Sample Buffer (Thermo Fisher, #NP0007) containing 2x NuPage Sample Reducing Agent (Thermo Fisher, #NP0009) by heating to 95 °C for 5 min. Proteins were separated on a 10% Criterion™ XT Bis-Tris Protein Gel (Biorad #3450112) and transferred to nitrocellulose membranes (Biorad #1620094). Membranes were incubated in a 5% powdered milk/TBS-0.5% Tween-20 blocking solution. The following antibodies were used to detect ALDOB and β-actin: α-Aldolase B (Sigma #SAB2108086, lot #QC12054) at 1:1000 in 5% Milk/TBS-T, α-Rabbit-HRP (Promega #401B) at 1:1000 in 5% Milk/TBS-T, and α-β-actin (Abcam #ab6276 Lot #Gr3207325-6) at 1:15,000 in 5% Milk/TBS-T, α-Mouse-HRP (Promega #402B) at 1:5,000 in 5% Milk/TBS-T. Protein bands were detected using Pierce ECL Western Blotting Substrate (Thermo Fisher, #32106) and the ChemiDoc MP Imaging System (BioRad #12003154). Blots were stripped with Restore PLUS Stripping Buffer (Thermo Fisher, #46428), washed, and blocked per the manufacturer's instructions in between probing with primary antibodies.

### 2.6 Plasma Biochemical analysis

Plasma biochemistry analysis was performed on the ADVIA Chemistry XPT (Siemens) for glucose (Siemens, #05001429), non-esterified FFAs (Randox Laboratories #FA115), triglycerides (Siemens, #10335892), and β-OHB (Randox Laboratories, #RB1008).

Insulin was measured using the Mouse/Rat Insulin Kit (Meso Scale Discovery #K152BZC).

### 2.7 Gene expression analysis

Liver RNA was extracted from liver tissue using the RNeasy Mini Kit (Qiagen, #74104) and RNA concentration was quantified using a NanoDrop 8000 (Thermo Fisher, #ND-8000-GL). 2 μg of RNA was reverse transcribed using the High-Capacity cDNA Reverse Transcription Kit (Applied Biosystems, #4368814) per the manufacturer's instructions except that the final volume for each reaction was 50 μL (final concentration was 40 ng/μL reaction). cDNA was diluted to 10 ng/μL with nuclease-free water (Ambion, #AM9937). For real-time PCR analysis, 20 ng of cDNA per reaction was mixed with TaqMan Gene Expression Master Mix (Applied Biosystems, #4369016) in a 384-well plate. The following TaqMan Gene Expression Assays were used (Applied Biosystems, #4331182): *Acaca* (Rn00573474_m1), *Acacb* (Rn00588290_m1), *Acadl* (Rn00563121_m1), *Acadm* (Rn00566390_m1), *Acads* (Rn00574634_m1), *Acox1* (Rn01460628_m1), *Aldob* (Rn01636758_m1), *Cpt1a* (Rn00580702_m1), *Cpt2* (Rn00563995_m1), *Fasn* (Rn00569117_m1), *G6pc* (Rn00689876_m1) *Hmgcl* (Rn00577753_m1), *Hmgcs2* (Rn00597339_m1), *Ldlr* (Rn005984442_m1), *Pcsk9* (Rn01416753_m1), *Pklr* (Rn01455286_m1), *Ppia* (Rn00690933_m1), *Scd1* (Rn00594894_g1), and *Slc2a5* (Rn00582000_m1). ChREBPα and β isoform mRNA transcripts were quantified using the Power SYBR green PCR master mix (Applied Biosystems, #4367659) with the following primer sequences (Invitrogen, #A15612), which were provided by Dr. Mark Herman: *Chrebpa* forward (5′-AGCATCGATCCGACACTCAC-3′), *Chrebpb* forward (5′-AGGTCCCAGGATCCAGTCC-3′), *Chrebpa*/*Chrebpb* universal reverse (5′-TGTTCAGCCGAATCTTGTCC-3′), and *Ppia* (IDT, #Rn.PT.39a.22214830). Real-time PCR was performed using The QuantStudio 7 Flex Real-Time PCR System (Applied Biosystems) using the following conditions: 50 °C for 2 min, 95 °C for 10 min, 95 °C for 15 s, and 60 °C for 1 min; the latter two steps were repeated for 40 cycles. Data was normalized to *Ppia* using the 2^−ΔΔCt^ method.

For RNAseq analysis RNA samples were prepared as described above. Prior to library construction, total RNA quality was assessed on a TapeStation 4200 (Agilent Technologies) with RNA ScreenTape reagents (Agilent Technologies) to generate RNA Integrity Number (RIN) scores. RIN scores for all samples were >7, exceeding the minimum input quality requirements for library construction. Total RNA mass was assessed on a Lunatic spectrophotometer (Unchained Labs) and a High Lunatic plate (Unchained Labs). For library construction, ∼300 ng of RNA was used. For samples with total RNA yield below this amount, all available RNA was used. Library construction proceeded through TruSeq Stranded mRNA Reference Guide (Illumina), omitting inline control DNA. Following library construction, libraries were qualified on a TapeStation 4200 instrument (Agilent Technologies) with D1000 ScreenTape reagents (Agilent Technologies). Total library mass was assessed via a high-throughput Qubit 1X dsDNA HS assay (Thermo Fisher Scientific) deployed on a CLARIOstar Plus fluorescent microplate reader (BMG Labtech). Libraries were normalized to 4 nM and equal volumes of each library were combined in a single pool. The resulting pool was sequenced on an NextSeq 500 (Illumina) using High Output (150 cycles) kit (Illumina), with 76 bp paired end reads. Sequencing data was processed and demultiplexed via bcl2fastq deployed on an internal system for handling next generation sequencing data.

### 2.8 Global gene expression profiling and pathway enrichment analysis

Raw RNA-seq FASTQ files were processed using count-based differential expression analysis to quantify gene expression. Sequencing reads were aligned to the rat genome (Rn6) using the STAR aligner (v.2.7.9) [14], followed by transcript quantification with Salmon (v1.10.1) [15]. Only transcripts with expression levels above a log2 TPM of 1.0 in at least three samples were considered expressed, with the rest filtered out, resulting in a set of 12,766 informative transcripts. Among these, only the protein-coding biotype of 12,127 genes was retained. Of these, 268 gene symbols lacked an associated Ensemble ID and were excluded from the analysis. Additionally, 120 Ensemble IDs associated with multiple gene symbols were also discarded. The final set of genes with valid Ensemble IDs consisted of 11,603 "background or detected genes."

The dataset included seven WT and six AldoB-KO rat liver samples under fasting conditions, and eight WT and eight AldoB-KO samples under fed conditions. Biological replicates for each condition (WT_Fed, AldoB-KO_Fed, WT_Fasted, AldoB-KO_Fasted) were pooled for differential expression analysis using DESeq2 [16].

To identify differentially regulated genes across the conditions, gene counts were modeled and transformed using DESeq2's default normalization procedures. Genes with an adjusted p-value ≤0.05 and an absolute log2 fold-change >1 were considered significantly differentially expressed. Reactome pathway enrichment analysis of differentially expressed genes (DEGs) was performed using the Bioconductor package enrichR (https://cran.r-project.org/package=enrichR).

### 2.9 Liver triglycerides

Approximately 25 mg of liver tissue was homogenized in 1 mL of cold triglyceride extraction buffer (10 mM Tris pH 7.4 and 0.1% Triton X-100 in 0.9% saline) using the TissueLyser II (Qiagen, #85300). Samples were centrifuged at 10,000 rpm for 2 min at 4 °C. The supernatant was diluted 2–3x in the extraction buffer and triglycerides were measured on the ADVIA Chemistry XPT (Siemens).

### 2.10 Liver DNL assessment by gas chromatography-mass spectrometry (GC–MS)

Rats were injected intraperitoneally with 20 mL/kg sterile deuterium oxide (Sigma, #151882) prior to an overnight fast. The next morning rats were euthanized; liver tissue was removed rapidly and flash frozen in liquid nitrogen. Approximately 50 mg of pulverized liver was homogenized in 1 mL of a 1:1 methanol:water solution in TissueLyser II (Qiagen, #85300). 500 μL of the liver lysate was transferred to a 10 mL glass vial. 875 μL of a 1:2 cholorform:methanol solution was added to the lysate, vortexed, and shaken at room temperature for 15 min 625 μL of chloroform containing 200 μM of glyceryl triheptadecanoate (Sigma Aldrich, #T2151) was added to the lysate and the sample was vortexed. 625 μL of a 0.9% sodium chloride solution (Sigma Aldrich, #S8776) was added and then the sample was centrifuged at 2,000 rpm for 15 min. The organic (lower) phase was transferred to a new glass vial and dried under a stream of nitrogen. 1 mL of 0.5 N NaOH in methanol was added to the dried residue, vortexed and heated at 90–95 °C for 20 min. The solution was then cooled on ice and 0.75 mL of 6 N HCl was added, followed by 1.75 mL of hexane. The sample was then vortexed, and the mixture was centrifuged at 1,200×*g* for 10 min. The organic (upper) phase was transferred to a new glass tube and dried under a stream of nitrogen. 400 μL of a 1:1 chloroform:methanol solution was added to the residue, followed by 100 μL of BF3 methanol solution (Sigma Aldrich, #15717), and then heated at 70 °C for 45 min. The solution was then cooled to room temperature. 500 μL of water was added to the sample followed by 1 mL of pentane; the sample was vortexed and then centrifuged at 2,000 rpm for 10 min. The organic (upper) layer was transferred to a new tube, dried under nitrogen, and reconstituted in 1 mL of hexane. The solution was then diluted 1:10 in hexane and analyzed by GC–MS on an Agilent 7000D triple quad mass spectrometer coupled to an Agilent 7890B Gas chromatograph. Separation was performed on an Agilent HP-5 ms column with initial temperature set at 100 °C which was held for 5 min then ramped at 7 °C/min until 225 °C was reached and held for an additional 5 min. Data was acquired using MS1 selected ion monitoring under positive chemical ionization with methane gas (methyl palmitate *m*/*z* 271, M+1 *m*/*z* 272, M+2 *m*/*z* 273, methyl heptadecaonoate *m*/*z* 285). Peak integration was performed with Agilent Mass Hunter software and calculations were performed as outlined (58).

### 2.11 Fructose, sucrose, and F1P quantification

Approximately 50 mg of liver tissue or 100 mg of diet were transferred into 1.5 mL Navy Eppendorf Lysis tubes (Next Advanced, #NAVYE1) and 500 μL of a pre-chilled 80% methanol/20% water (Fisher Scientific, #A456-4 and #W64) solution was added. Samples were homogenized in the Bullet Blender Tissue Homogenizer Storm Pro (Next Advanced). Samples were centrifuged at 15,000×*g* for 2 min at 4 °C and an internal standard, ^13^C-6 fructose (Cambridge Isotopes, #CLM-1553), D-sucrose (^13^C-6 glucose) (Cambridge Isotopes, # CLM-8091), or D-glucose-D7 (Cambridge Isotopes, #DLM-2062) was added for a final concentration of 100 μM upon reconstitution and vortexed. Samples were centrifuged at 15,000×*g* for 10 min at 4 °C and the supernatant was transferred to new 1.5 mL tubes and the centrifugation was repeated. Samples were transferred to a 96-well plate (Waters, #186005837) and dried down under nitrogen. Samples were reconstituted in 70% acetonitrile/80 % methanol (Fisher Scientific, #A955-4) containing ^13^C-6 fructose and F1P (Sigma, #F1127) as external standards. LC-MS analysis was performed on a Q Exactive Plus Orbitrap (Thermo Fisher) coupled with the Dionex UltiMate 3000 RSLCnano System (Thermo Fisher). The mass spectrometer settings were as follows: source fragmentation: none; sheath gas flow rate: 45; auxiliary gas flow rate: 15; sweep gas flow rate: 3; spray voltage: 3.00 kV; capillary temperature: 310 °C; S-lens RF level: 50; auxiliary gas heater temperature: 350 °C; scan range: 65.0–975.0 *m*/*z*; resolution: 140,000; negative polarity; AGC target: 3 × 10^6^; Maximum injection time: 500 ms. The liquid chromatography was performed with a HILICpak VT-50 2D column (150 mm × 2.0 mm, 5 μm particle size, Shodex #F7630400) using Buffer A (90% acetonitrile and 10% water which contains 20 mM 1:1 triethylamine:formic acid at pH 9.18) and Buffer B (5% acetonitrile and 95% water which contains 54 mM 1:1 triethylamine:formic acid at pH 3.03). To analyze the diet samples the following settings were used: 0% B for 10 min, increased up to 16% B for 17 min, increased up to 65% for 5 min, 87% B for 2 min, 100% B for 13 min, and then 0% B for 13 min with a flow rate of 0.2 mL/min for 5 min, 0.3 mL/min for 53 min, and 0.2 mL/min for 2 min. To analyze the liver samples the following settings were used: 0% B for 6 min, increased up to 13% B for 21 min, 20% B for 2 min, 100% B for 4 min, and 0% B for 6 min with a flow rate of 0.3 mL/min. Peaks were integrated using AreaTop with a 5 ppm mass tolerance in MAVEN (774, Princeton University) and metabolite concentrations were calculated using external standard curves made from ^13^C to 6 fructose (0–600 μM) or F1P (0–200 μM) using KaleidaGraph (3.50, Synergy Software).

### 2.12 Hepatic metabolite analysis

Human Metabolome Technologies, Inc. profiled hepatic metabolites using CE-TOFMS and CE-QqQMS for cation and anion analysis, respectively. Peaks were integrated using MasterHands (ver.2.18.0.1, Keio University) and MassHunter Quantitative Analysis (B.06.00, Agilent Technologies). Metabolites were quantified by normalizing data to internal standards. Samples with abnormally high or low metabolites were excluded from analysis.

Quantified metabolites were analyzed using MetaboAnalyst 6.0, one factor analysis [17,18]. Missing values were replaced by 1/5 of the minimum positive value of a given metabolite concentration which was assumed to be the limit of detection. The data was log10 transformed and pareto scaled. A volcano plot was generated and metabolites with a fold change threshold of +/−1.5 and a p-value threshold of >0.05 were identified as different.

### 2.13 Primary hepatocytes

Freshly isolated primary hepatocytes from WT and AldoB-KO rats (Biomere, Worcester, MA) were seeded at a density of 1∗10^4^ cells/well onto XF96 V3 PS Cell Culture Microplates (Agilent Technologies, #101085-004), pre-coated with Rat Tail Collagen Coating Solution (Sigma #122-20) at 1 μg/well for the Seahorse assays or at 2.5∗10^5^ cells/well onto 24-well plates (Falcon, #353047), precoated at 10.5 μg/well for the glucose production assay, in high-glucose DMEM (Gibco #11995) supplemented with 10% FBS (Gibco #16140) and 1% Pen/Strep (Gibco #15140) for 4 h. Media was refreshed and supplemented further with 100 nM dexamethasone (Sigma #D4902) and 100 nM insulin (Seahorse) or 1 nm insulin (glucose production) (Sigma #I9278) and cells were cultured overnight. The following morning, cells were assayed with the XF Long Chain Fatty Acid Oxidation Stress Test Kit assay (Agilent Technologies, #103672-100) or the XF Palmitate Oxidation Stress Test Kit assay (Agilent Technologies, #103693-100) per the manufacturer's instructions, except the cells were cultured in the substrate-limited growth media for 4 h instead of overnight. The final concentration of FCCP was 2 μM for both assays. For the glucose production assay, cells were washed and incubated in Krebs media (110 mM NaCl, 4.7 mM KCl, 1.2 mM MgSO_4_, 1.2 mM KH_2_PO_4_, 20 mM NaHCO_3_, 10 mM HEPES, 1.2 mM CaCl, and 0.1 % BSA; pH adjusted to 7.4) supplemented with 1 μM glucagon (Sigma #G2044) for 2 h. The media was replaced and supplemented with 10 mM fructose, 5 mM glycerol, 1:10 (2:20 mM) pyruvate/lactate, or 10 mM xylitol. After 5 h, media was collected, and glucose was measured using the microtiter protocol for the Autokit Glucose Reagent (Wako #997–03001). Following the assays, cells were lysed in RIPA Lysis and Extraction Buffer (Thermo Fisher #89900), supplemented with cOmplete mini EDTA-free protease inhibitor cocktail tablets (Sigma #11836170001) and PhosSTOP tablets (Sigma #PHOSS-RO). Protein was measured as described above. Data was normalized to protein content.

### 2.14 Histological analysis

Sections of the right lateral, left lateral, and right medial lobes were placed in an embedding cassette, immersed in 10% buffered formalin, and processed to paraffin blocks using standard tissue processing protocols for histology. Formalin-fixed paraffin-embedded liver sections were used for H&E and Picrosirius red (PSR) histochemical staining. Sections were cut using a rotary microtome and mounted on glass slides. Five-micron thick sections were prepared and routinely stained with H&E; 7-μm thick sections were prepared for PSR staining. For PSR (Rowley Biochemical Inc., # F-357-2), slides were stained using the Tissue-Tek Prisma® Plus Automated Slide Stainer (Sakura Finetek USA Inc). Slides were deparaffinized and placed overnight in Bouin's Fluid before returning to the stainer and stained as per manufacturer's protocol with some optimized steps (1 % Phosphomolybdic Acid for 5 min; 0.1 % Sirius Red in Saturated Picric Acid for 90 min; 2 × 30 s wash in 0.5 % Acetic Acid). Slides were automatically dehydrated and then mounted with a permanent mounting medium. H&E and PSR stained sections of liver tissues were evaluated by a board-certified veterinary pathologist.

### 2.15 HFI and MASH association analysis

Three longitudinal, US-based electronic health record (EHR) and claims healthcare databases were used: the Optum® Humedica EHR database, the IBM MarketScan Commercial Claims and Encounters and Medicare Supplemental (CCAE/MDCR) Commercial claims database, and the IQVIA PharMetrics Plus claims database. Patients were included in the study in each data source if they had at least one healthcare encounter during the study period, between January 1, 2017 and December 31, 2019. Patients were selected as a case (numerator) in the study if the following inclusion criteria were met: at least one inpatient diagnostic code or two outpatient diagnostic codes that were 30 days apart. The following diagnostic codes from The International Classification of Diseases, 10th edition (ICD-10) were used: HFI: E74.12, NASH: K75.81, Acute lymphoid leukemia (ALL): C91.00, C91.01, C91.02, cervical dystonia: G24.3, epilepsy: G40, and multiple sclerosis (MS): G35.2x2 tables were generated from each database for HFI versus the other diseases. Note, at the time of analysis MASH was referred to as Nonalcoholic Steatohepatitis (NASH) so all queries were made against NASH.

### 2.16 Statistics

The 2x2 tables were analyzed as previously described by Desai et al. [10] using R (v4.0.0; https://www.R-project.org/). Briefly, Fisher's exact test (or logistic regression where appropriate) was used to calculate whether the relationship was statistically significant. Cochran's *Q* test was used to determine heterogeneity and a fixed-effects meta-analysis was used. The difference of OR between HFI versus MASH and HFI versus negative controls (ORR) was then calculated. Data for the HFI and MASH association is presented as the log(OR) and 95% CI.

Graphical data are presented as means ± SEM. ANOVA with Tukey's post-hoc test was used to determine the differences across multiple groups. Two-tailed t-test was used to determine differences between two groups. The ROUT method was used to identify outliers in each data set. Statistics were calculated using GraphPad Prism 9 (GraphPad).

## 3 Results

### 3.1 AldoB-deficient rats recapitulate human HFI

**Figure 1:**
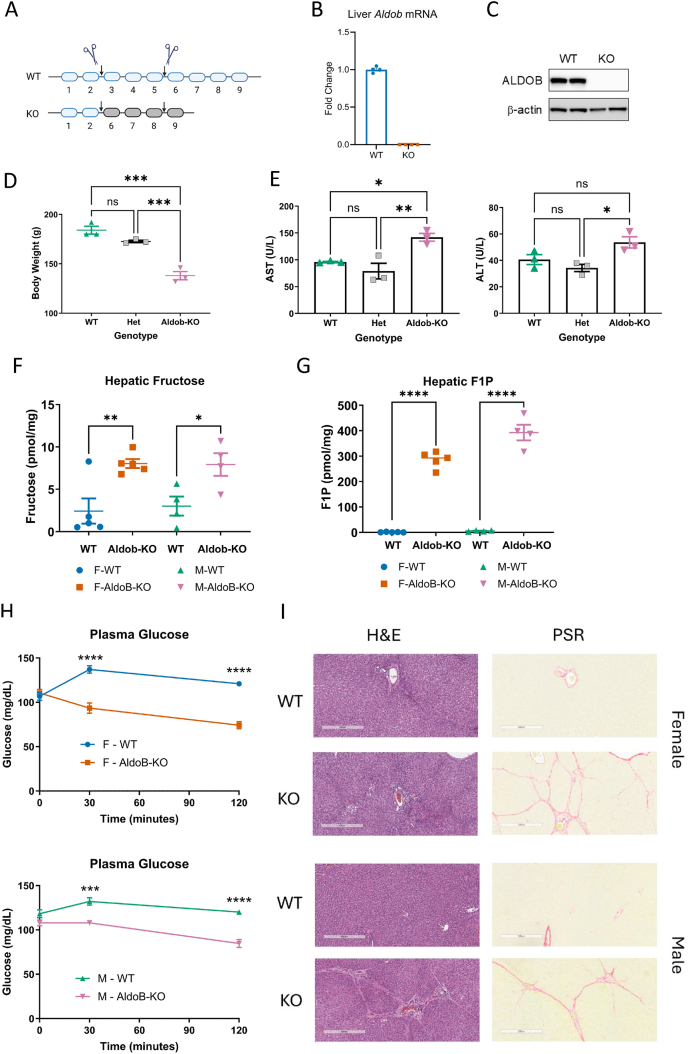
**Aldob-KO rats phenocopy HFI traits.** (**A**) Schematic of CRISPR/Cas9-mediated deletion of exons 3–5 in the WT rat *Aldob* gene to produce the Aldob-KO (KO) allele. (**B**) mRNA expression of *Aldob* in the liver of WT and Aldob-KO (KO) rats (12–13 weeks). (**C**) Western blot ALDOB protein expression in the liver of WT and KO rats (12–13 weeks). (**D**) Body Weight and (**E**) plasma AST and ALT levels of WT, Heterozygote (HET) and AldoB-KO rats, 8–9 weeks of age and weaned on diet 2920X containing 0.15% fructose. Fasting hepatic (**F**) fructose and F1P (**G**) levels in 11–13 week old male and female WT and AldoB-KO rats. (**H**) Plasma glucose in female (top) and male (bottom) measured over time in 11–13 week old WT and AldoB-KO rats orally administered fructose. (**I**) Liver histological sections stained with hematoxylin and eosin (H&E) and picrosirius red (PSR) from WT and AldoB-KO rats weaned on diet 2920X for 5 weeks and then switched to D16121902 for an additional 18 weeks. Schematic in **A** created in BioRender. Tesz, G. (2025) https://BioRender.com/28oxdca. Statistical significance determined by one-way ANOVA with Tukey's multiple comparisons test except for **H** where two-way ANOVA with Tukey's multiple comparisons test was used. ∗p < 0.05 ∗∗p < 0.01 ∗∗∗p < 0.001 ∗∗∗∗p < 0.0001.

To determine if fructose independent metabolic dysfunction exists in HFI exons 3–5 of *Aldob* were deleted in rats using CRISPR (Figure 1A), resulting in ablation of hepatic *Aldob* mRNA and ALDOB protein (Figure 1B,C). In initial studies, AldoB-KO rats weaned on a commercially available fructose-free diet (2920X) weighed significantly less than WT rats (Figure 1D), an observation consistent with unintended fructose exposure in children with HFI [9]. We measured the fructose content of diet 2920X using LC-MS [19] and discovered that fructose was indeed present (0.15% w/w). Fructose, at these trace amounts increased liver enzymes in AldoB-KO rats confirming their sensitivity to the carbohydrate (Figure 1E). An alternate diet, D16121902, was verified to be virtually fructose-free (0.02% w/w fructose) and used in all studies going forward unless otherwise noted. When fed D16121902, body weight, liver enzymes and hepatic triglycerides were similar between genotypes of both sexes (Supplemental Fig. 1A–1D). Liver morphology was grossly normal except for a slight increase in vacuolation observed in AldoB-KO rats (Supplemental Fig. 1E).

To confirm the absence of ALDOB activity, hepatic F1P and fructose levels were first measured in fasted male and female AldoB-KO rats. Consistent with inhibition of fructose metabolism, elevated hepatic fructose and F1P were observed in AldoB-KO rats irrespective of sex compared to WT littermates (Figure 1F,G respectively). In a separate study, fructose was administered orally and plasma glucose was monitored over time in AldoB-KO and WT rats, as hypoglycemia has been reported in HFI subjects following fructose administration [9,[20], [21], [22]]. In both male and female WT rats, plasma glucose increased following the fructose bolus due to the conversion of fructose to glucose via unimpeded fructose metabolism [23]. However, in AldoB-KO rats, glycemia decreased in both sexes further confirming impaired fructose metabolism (Figure 1H). Finally, since early fructose exposure in children with HFI causes hepatic fibrosis, often persisting for years after dietary fructose removal, we also investigated the effects of weaning rats from diet 2920X to D16121902 to understand the effects of early fructose exposure. In this study rats were initially fed 2920X for 5 weeks and then switched to D16121902 for 18 additional weeks and allowed to recover. Both male and female AldoB-KO rats had evident hepatic fibrosis despite switching from diet 2920X to diet D16121902 (Figure 1I). Thus, AldoB KO rats exposed to trace fructose replicated multiple human HFI features, confirming the model's suitability for studying HFI.

Aldolase B is also expressed in the intestines and kidney which contributes to systemic glucose and lipid metabolism. Gross examination of both organs did not reveal any abnormalities in our studies. Histological examination of kidney sections was also conducted from rats fed D16121920 or 2920X for 4 weeks and revealed that the kidneys were mostly normal between genotypes and genders regardless of diet (Supplemental Table 1). However, female AldoB-KO rats had mild evidence of minimal dilation/hypertrophy of the proximal tubules (P3 segment) located in the corticomedullary junction that was greater than in the WT female rats. Given that the changes in the kidney were unremarkable, we decided to focus our studies on the effects of ALDOB deletion in the liver.

### 3.2 Fasted AldoB-KO rats have altered hepatic lipid metabolism in the absence of fructose

**Figure 2:**
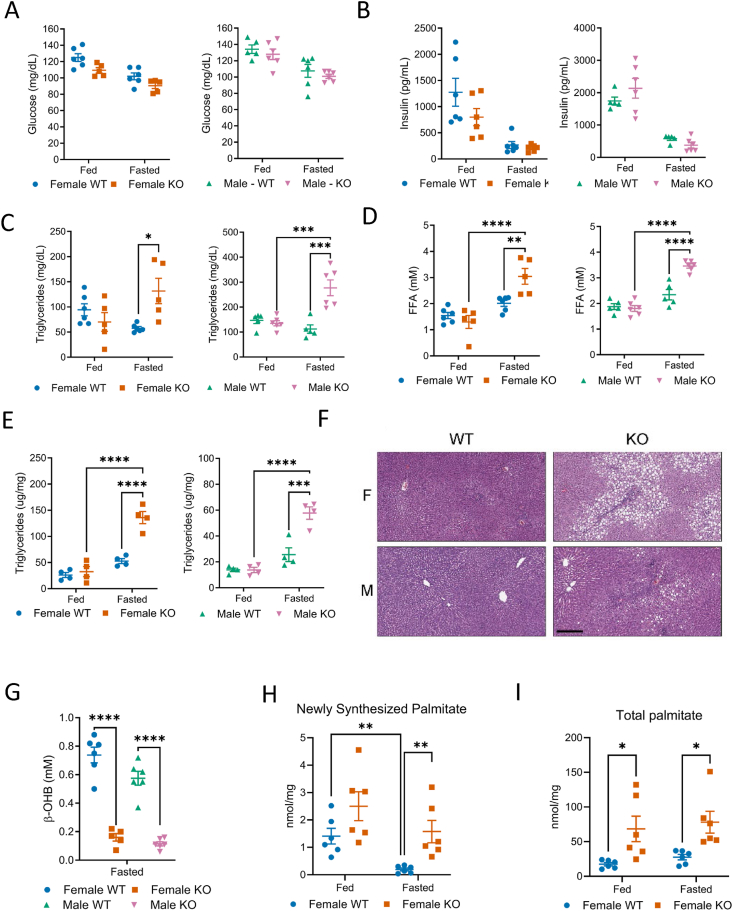
**AldoB-KO rats develop steatosis and hyperlipidemia when fasting.** Fed and fasted (**A**) plasma glucose and (**B**) plasma insulin in 16-18-week-old WT and KO rats. (**C, D**) Fed and fasted plasma triglycerides and FFA in 16-18-week-old rats WT and KO rats. (**E**) Fed and fasted hepatic triglycerides in 23-week-old WT and KO rats. (**F**) Representative H&E images of livers from 11 to 13-week-old fasted WT and KO rats. Scale bar = 300 μm. (**G**) Fasted plasma β-OHB in WT and KO rats. (**H, I**) Newly synthesized and total palmitate in 29-week-old fed and fasted rats injected with deuterium oxide prior to fasting. Statistical significance determined by one-way ANOVA with Tukey's multiple comparisons test except for H and I where two-way ANOVA with Tukey's multiple comparisons test was used. ∗p < 0.05 ∗∗p < 0.01 ∗∗∗p < 0.001 ∗∗∗∗p < 0.0001.

As discussed previously, ALDOB facilitates liver carbohydrate catabolism and anabolism dependent on nutritional status. Therefore, the effect of *Aldob* deletion on glycemic endpoints was measured. In fed rats, plasma glucose was similar between genotypes and decreased slightly upon fasting in rats of both sexes (Figure 2A). Though not statistically significant, decreased plasma glucose levels were observed in male and female AldoB-KO rats in both nutritional states (Figure 2A). Insulin concentrations were also similar between male and female rats of both genotypes and decreased to a similar extent upon fasting (Figure 2B).

Whether loss of *Aldob* also altered plasma and hepatic lipids was explored next. In fed rats, plasma triglycerides and free fatty acids were similar between genotypes (Figure 2C,D). Upon fasting both plasma lipid species increased significantly in male and female AldoB-KO rats (Figure 2C,D). Hepatic triglycerides were also similar in fed WT and AldoB-KO rats of each gender. Consistent with changes in AldoB-KO rat plasma triglycerides, elevated hepatic triglycerides were observed in AldoB-KO rats of both sexes compared to WT (Figure 2E,F).

Hepatic triglyceride levels reflect several metabolic processes including hepatic FAOx, DNL and peripheral lipid uptake [24]. Additionally, FFA levels reflect a balance between utilization and production. Since ALDOB is not expressed in adipocytes [2,3] we reasoned that the increase in FFA (Figure 2D) could be due to a hepatocyte-specific metabolic defect in FA utilization. In agreement, circulating β-hydroxybutyrate (β-OHB), a product of hepatic FAOx [25], decreased signigicantly in fasted AldoB-KO rats relative to WT confirming impared hepatic fatty acid oxidation (Figure 2G). This result is consistent with reports of reduced levels of fasted β-OHB in subjects with HFI [26]. DNL was assessed by measuring the incorporation of ^2^H into palmitate in fed and fasted AldoB-KO and WT female rats. Measurement of newly synthesized palmitate reflected the nutritional status in WT rats as fasting decreased ^2^H-labeled palmitate in WT rats (Figure 2H). In contrast to WT rats, newly synthesized palmitate levels remained elevated in fasted AldoB-KO rats (Figure 2H). Total hepatic palmitic acid levels were also 3–4 -fold higher in fed and fasted AldoB-KO rats (Figure 2I). Thus, the steatosis and hyperlipidemia observed in AldoB-KO rats could likely be due to impaired regulation of hepatic lipid metabolism.

### 3.3 Altered glycolytic and fatty acid metabolic gene expression in fasted AldoB-KO livers

**Figure 3:**
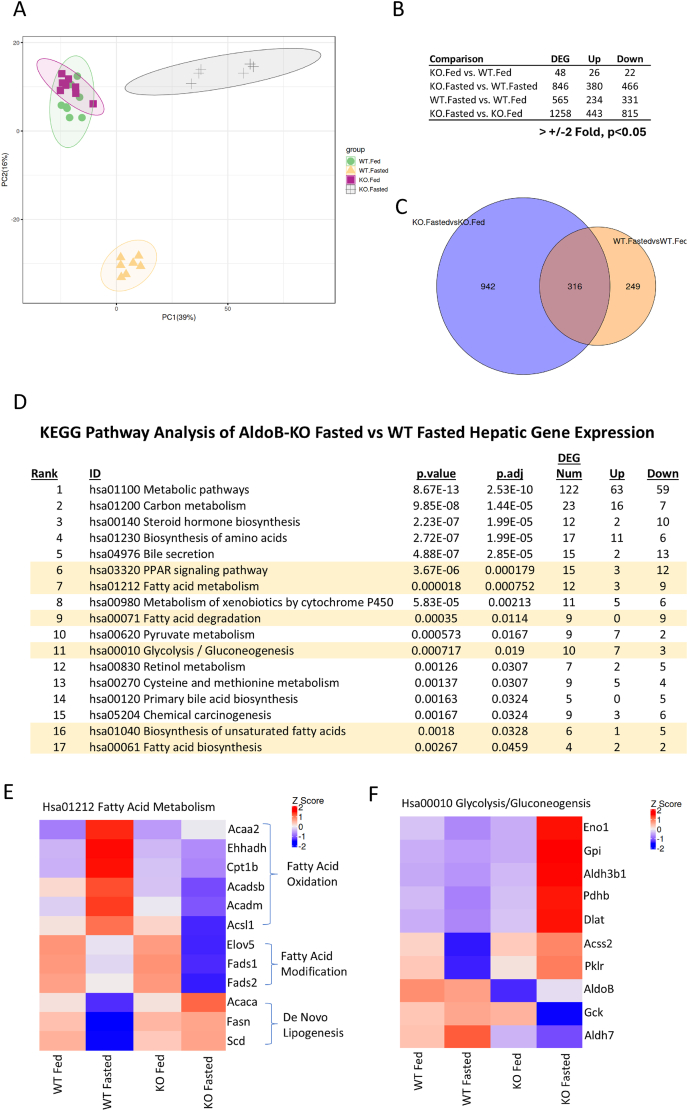
**Fasting increases lipogenic and glycolytic gene expression in AldoB-KO rats.** (**A**) Principal component analysis of differentially expressed genes in 11-week old, male WT and AldoB-KO rats in the fed and fasted state as measured by bulk RNAseq analysis. (**B**) Differential expressed genes (DEG) up-regulated and down-regulated with greater than +/− 2-fold change in expression and p < 0.05. (**C**) Venn diagram showing the number of DEGs regulated in fasted AldoB-KO and WT rats and the number of genes similarly regulated between genotypes. (**D**) KEGG pathway analysis of DEGs identified by RNAseq and subsequent pathways enriched in DEGs. Pathways involved with fatty acid or glucose metabolism are highlighted. Heat map showing expression levels of genes in the KEGG pathways for fatty acid metabolism (**E**) and glycolysis/gluconeogenesis (**F**).

To understand the mechanism by which loss of *Aldob* alters hepatic lipid metabolism, bulk RNAseq analysis was performed on fed and fasted male WT and AldoB-KO rat livers. Principal component analysis revealed similar hepatic gene expression between fed WT and AldoB-KO rats (Figure 3A) with only 48 genes differentially expressed (figure 3B). Upon fasting, WT and AldoB-KO hepatic gene expression differed substantially (Figure 3A) with 846 genes differentially expressed (Figure 3B). A total of 565 and 1258 genes were expressed differently in fasted WT and AldoB-KO livers respectively when compared to fed livers (Figure 3B) with only 316 genes regulated similarly between genotypes (Figure 3C).

To understand which pathways were dysregulated in fasted AldoB-KO livers, KEGG Pathway Analysis was performed. Seventeen pathways primarily involved with lipid and central carbon metabolism had significant differences in hepatic gene expression (Figure 3D). Pathways involved with lipid metabolism were first explored because of the observations in fasted AldoB-KO rats. In the fed livers, genes regulating fatty acid metabolism were similar between genotypes (Figure 3E). As expected, fasting decreased DNL related genes (*Acaca, Fasn* and *Scd*) in WT livers while the expression of genes facilitating fat oxidation (*Ehhadh, Acadm, Acsl1 etc*) increased. Surprisingly, most of these genes displayed the opposite expression pattern in fasted AldoB-KO livers. Genes required for DNL increased in expression while FAOx related genes decreased relative to fed KO livers (Figure 3E).

Expression of genes regulating glycolysis and gluconeogenesis also were differentially expressed in fasted AldoB-KO rat livers relative to WT (Figure 3D). Like our observations with genes regulating lipid metabolism, genes associated with glycolysis had similar expression levels in fed AldoB-KO and WT rats (Figure 3F). Upon fasting glycolytic/gluconeogenic gene expression was substantially different in WT and AldoB-KO rat livers. The expression of many glycolytic genes decreased in wild-type fasted rat livers, while in AldoB-KO livers, most glycolytic genes increased significantly (Figure 3F). Notably, many of the glycolytic (*Pklr, Acss2, Gpi* and *Eno1*) and lipogenic genes (*Acaca*, *Fasn* and *Scd*) found elevated in fasted AldoB-KO livers are transcriptional targets of the carbohydrate response element binding protein (ChREBP) [27]. In addition to promoting DNL, ChREBP overexpressed via adenovirus, has been demonstrated to suppress FAOx in mice [28]. Thus, inappropriate activation of ChREBP may drive DNL and suppress FAOx in fasted the *Aldob*−/− rats.

**Figure 4:**
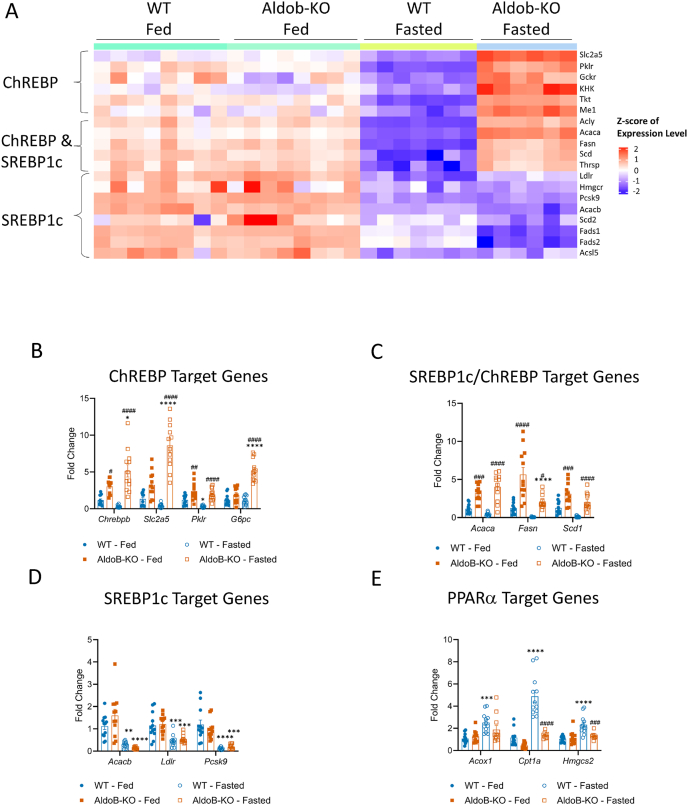
**Increased ChREBP target gene expression in AldoB-KO rats upon fasting.** (**A**) Heat map comparing ChREBP, SREBP1c and dual (ChREBP/SREBP1c) target gene regulation in 11-week-old male fed and fasted WT and AldoB-KO rats. Targeted qrtPCR expression analysis of hepatic gene targets of ChREBP (**B**), ChREBP/SREBP1c (**C**), SREBP1c (**D**), and PPARα (**E**) from 23 to 32-week-old fed and fasted, female, WT and KO rats, normalized to fed WT controls from two different studies combined. Two-way ANOVA with Tukey's multiple comparisons test was used to calculate statistical significance. ∗p < 0.05 ∗∗p < 0.01 ∗∗∗p < 0.001 ∗∗∗∗p < 0.0001 vs fed; #p < 0.05 ##p < 0.01 ###p < 0.001 ####p < 0.0001 vs WT.

Sterol regulatory element binding protein 1c (SREBP1c) and ChREBP transcriptionally regulate many lipid metabolizing enzymes [27,[29], [30], [31], [32]]. Additionally, ChREBP also regulates genes involved with fructose catabolism and glycolysis [13,27]. Thus we compared the expression of genes regulated by SREBP1c, ChREBP or both transcription factors to determine if one or both contributed to the lipid phenotype. As expected, the expression of genes regulated by SREBP1c and ChREBP decreased in fasted WT livers (Figure 4A). In AldoB-KO livers, fasting suppressed SREBP1c target genes similarly to WT rats. However, mRNA levels of ChREBP-regulated genes were significantly higher in fasted AldoB-KO rat livers than in the fed state (Figure 4A), indicating increased ChREBP activity. Elevated ChREBP target gene expression was confirmed by qRT-PCR in livers from fasted female (Figure 4B,C) and male (Supplemental Fig. 2A and B) WT and AldoB-KO rats in separate studies. Normal regulation of SREBP1c target gene expression was also confirmed with targeted gene expression analysis in livers of both genotypes and sexes (Figure 4D and Supplemental Fig. 2C).

Our pathways analysis also identified other dysregulated transcriptional pathways important for lipid metabolism in fasted AldoB-KO rats including peroxisome proliferator-activated receptor alpha (PPARα), a transcriptional regulator important for FAOx and ketogenesis [33] (Figure 3D). Most PPARα transcriptional targets decreased upon fasting in AldoB-KO rat livers relative to WT (Supplementary Table 2). Though the PPARα target genes *Mei* and *Scd*, also co-regulated by ChREBP [27], were increased in AldoB-KO livers (Supplementary Table 2). Targeted transcriptomic analysis in a separate study also confirmed suppression of PPARα target genes in fasted AldoB-KO livers (Figure 4E female and Supplemental Fig. 2D male). Additionally, the expression of genes regulating bile acid metabolism and steroid hormone biosynthesis were also differentially regulated in fasted AldoB-KO livers with many downregulated genes (Figure 3D and Supplementary Table 3).

In total the transcriptomics data demonstrates inappropriate regulation of hepatic genes that regulate DNL, FAOx, glycolysis/gluconeogenesis and bile acid metabolism in the rats lacking *Aldob*. The increase in ChREBP target gene expression could potentially drive hyperlipidemia and steatosis observed in AldoB-KO rats.

### 3.4 Glycolytic metabolites accumulate in fasted AldoB-KO livers

**Figure 5:**
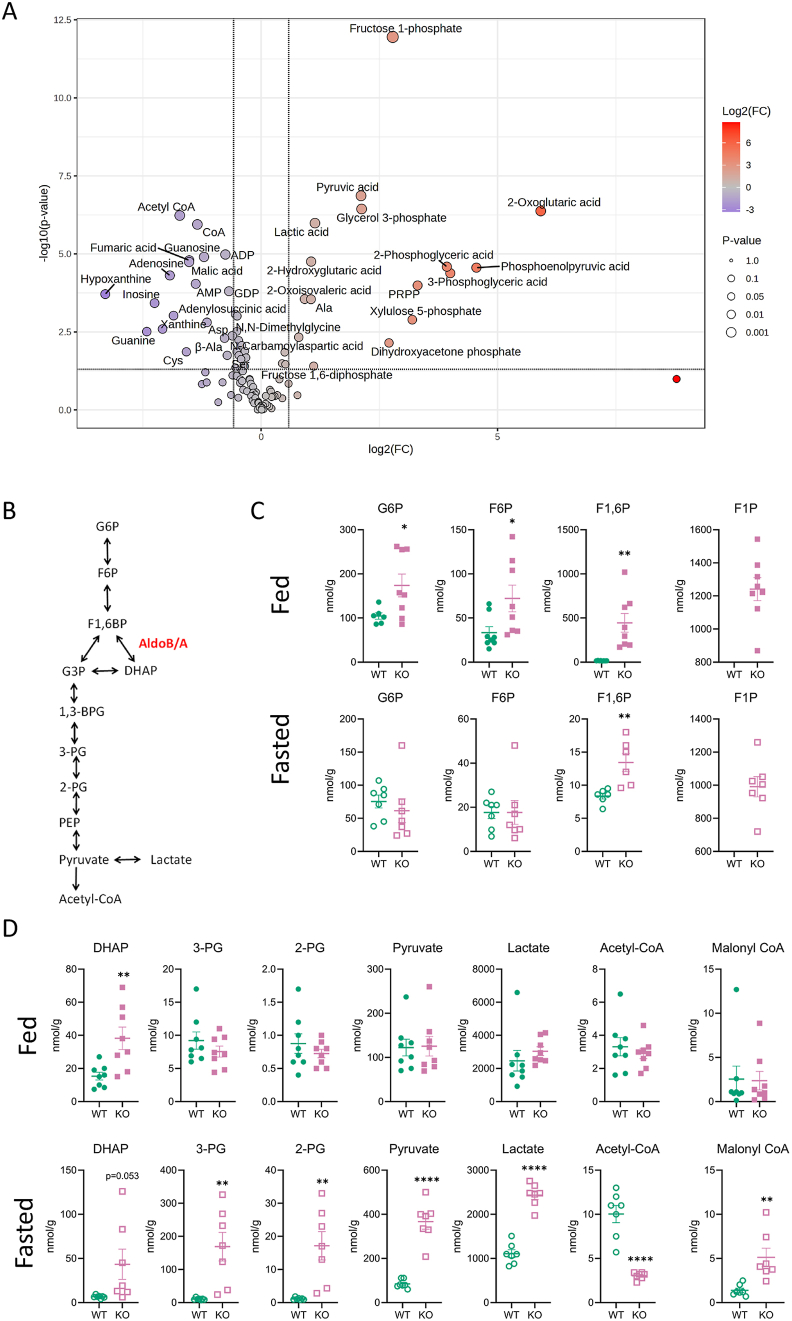
**Glycolytic metabolites accumulate in fasted AldoB-KO livers.** (**A**) Volcano plot showing metabolites that are significantly different (>1.5 fold different vs WT and p value < 0.05) between fasted WT and AldoB-KO livers from 11-week-old rats. (**B**) Schematic showing the glycolytic and gluconeogenic metabolites and the aldolase reaction. (**C**) Fed and fasted levels of sugar phosphates. (**D**) Three and two-carbon glycolytic metabolites and glycolytic products. Statistical significance determined by unpaired t test. ∗p < 0.05, ∗∗p < 0.01, ∗∗∗p < 0.001, ∗∗∗∗p < 0.0001 vs WT.

As discussed above, Aldolase B is an essential enzyme for fructolysis, glycolysis and gluconeogenesis (Figure 5B) [7,8]. We hypothesized that the absence of Aldolase B during fasting may impair hepatic gluconeogenesis, leading to an accumulation of glycolytic intermediates, potentially increasing in ChREBP activity and transcriptional target genes (Figure 4). To test this hypothesis, global metabolomics was performed on fed and fasted AldoB-KO rat livers and compared to WT. In fed livers only 10 metabolites were differentially enriched in AldoB-KO compared to WT rat livers (Supplemental Fig. 3A and Supplementary Table 4). These metabolites included F1P, despite being on a diet virtually devoid of fructose, and other glycolytic (F1,6P, F6P, G1P, G6P and DHAP), amino acid (proline, alanine, sarcosine and cystathionine) and pentose phosphate (erythrose 4-phosphate) metabolites (Figure 5C and Supplementary Table 4). In contrast to the fed state, 35 metabolites found in glycolytic, amino acid, tricarboxylic acid cycle (TCA), pentose phosphate and other metabolic pathways were dysregulated in fasted AldoB-KO livers (Figure 5A and Supplementary Table 5). As in the fed state, F1P was significantly upregulated in fasted AldoB-KO livers compared to WT (Figure 5C and Supplemental Table 5). Relative to WT, hepatic hexose phosphate concentrations, G6P and F6P, were elevated to a greater extent in Aldo-B KO rats during the fed state compared to fasting (Figure 5C). In the fed state, F1,6P levels in AldoB-KO livers were 29.5 times higher than controls (445.3 vs 15.1 nmol/g). During fasting, F1,6P levels decreased substantially in AldoB-KO livers and were only 1.6 times higher versus WT (13.4 vs 8.4 nmol/g). Overall, the levels of F1,6P returned to near WT levels in the fasted livers suggesting that elevated F1,6P was predominantly a feature of fed hepatic metabolism in AldoB-KO rats.

Fasting also increased metabolites downstream of the aldolase reaction as hepatic concentrations of DHAP, 3-PG, 2-PG, Pyruvate and Lactate were all elevated in AldoB-KO rat livers (Figure 5D and Supplemental Table 5). Acetyl-CoA levels increased by ∼3 fold upon fasting in WT but not in AldoB-KO livers. Acetyl-CoA is generated from both fatty acid oxidation and glycolysis and TCA metabolism. Additionally, carboxylation of acetyl-CoA by ACC1/2 (product of *Acaca/Acacb*) generates malonyl-CoA, an essential substrate for DNL and an inhibitor of FAOx [34,35]. The 3-fold reduction in Acetyl-CoA levels between AldoB-KO and WT rat livers could be due to an impairment in fatty acid oxidation and an elevation of DNL [[34], [35], [36]] (Figure 2G,H and I). Thus, we quantified malonyl-CoA in AldoB-KO and WT livers using a separate LC-MS method and found the levels of the metabolite increased in fasted, but not in fed, AldoB-KO rat livers compared to WT (Figure 5D). Changes were also observed in the energetic nucleotides ADP/AMP, pentose phosphate, TCA, amino acids, and other metabolites (Supplemental Table 5). Thus, we suspected that the elevations in malonyl-CoA in fasted AldoB-KO rat livers may impair fatty acid oxidation and enhance DNL.

### 3.5 Glycolysis and fatty acid oxidation are impaired in AldoB-KO hepatocytes

**Figure 6:**
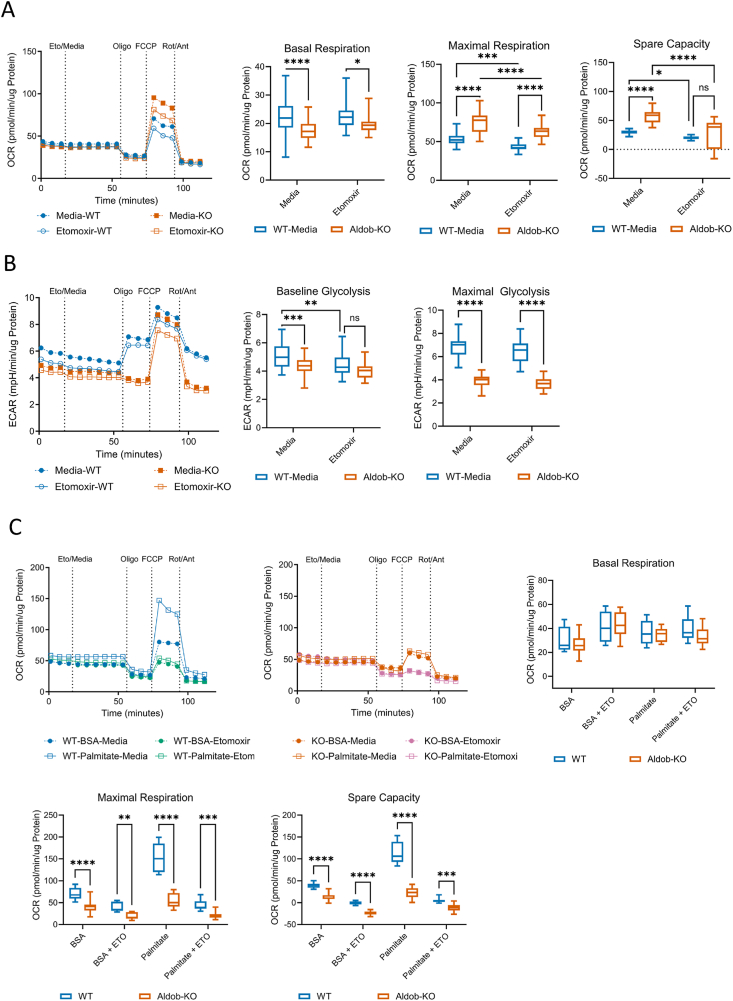
**Long-chain fatty acid oxidation is impaired in Aldob-KO hepatocytes.** (**A**) Representative OCR plot of WT and KO hepatocytes cultured in 10 mM glucose (n = 18–22) and min and max box and whisker plots of basal respiration, maximal respiration, and spare capacity of WT and KO hepatocytes cultured in 10 mM glucose (n = 31–44 total from two experimental replicates). (**B**) ECAR plot from representative study in panel A and baseline glycolysis and maximal glycolysis calculation. (**C**) Representative OCR plot of WT or KO hepatocytes cultured in 0.5 mM glucose, with or without palmitate (n = 8–12) and min and max box and whisker plots of basal respiration, maximal respiration, and spare capacity (n = 15–21 total from two experimental replicates). Statistical significance determined by 2-way Anova with Sidak's test for multiple comparisons. ∗p < 0.05 ∗∗p < 0.01 ∗∗∗p < 0.001 ∗∗∗∗p < 0.0001.

The alterations in glycolytic metabolites indicated a change in glycolytic metabolism in fasted AldoB-KO rats. Therefore, Seahorse technology was employed to examine glycolytic and mitochondrial activity in isolated AldoB-KO hepatocytes. First, mitochondrial function was assessed by measuring oxygen consumption under standard complete nutrient conditions (10 mM glucose). Under these conditions, AldoB-KO hepatocytes had a mostly normal respiratory profile despite having a slightly lower basal respiration compared to WT hepatocytes (Figure 6A). Treatment with the uncoupler FCCP increased oxygen consumption in both genotypes as anticipated. However, a higher maximal respiration, and a greater spare capacity was observed in AldoB-KO compared to WT hepatocytes. This increase in spare capacity and maximal respiration suggests that mitochondrial capacity is not limited in the AldoB-KO hepatocytes. Since ALDOB participates exclusively in fructolysis and glycolysis, minimal changes in OCR were anticipated in AldoB-KO hepatocytes. Additionally, the increase in mitochondrial capacity could be compensatory for decreased glycolytic capacity in AldoB-KO hepatocytes.

Since fatty acid oxidation was impaired in fasted AldoB-KO rats, isolated hepatocytes were incubated with the CPT1 inhibitor Etomoxir to assess lipid utilization in nutrient replete conditions. The addition of Etomoxir did not affect basal respiration in hepatocytes respective to their genotype, though reduced basal respiration was still observed in AldoB-KO versus WT hepatocytes (Figure 6A). With Etomoxir treatment, FCCP stimulated oxygen consumption was slightly reduced in both WT and AldoB-KO hepatocytes indicating that hepatocytes of both genotypes utilize fatty acids in the nutrient replete setting. As observed without CPT1 inhibition, both maximal respiration and spare capacity were enhanced in AldoB-KO versus WT hepatocytes with Etomoxir treatment, demonstrating enhanced oxidative capacity in AldoB-KO hepatocytes.

While assessing mitochondrial function, glycolysis was also interrogated in WT and AldoB-KO hepatocytes by measuring the extracellular acidification rate (ECAR). At baseline, a reduction in ECAR was observed in AldoB-KO hepatocytes compared to WT under standard conditions (Figure 6B). Treatment with the ATP synthase inhibitor oligomycin increased ECAR in WT hepatocytes to compensate for inhibition of oxidative phosphorylation (Figure 6B). Interestingly, in AldoB-KO hepatocytes ECAR did not change with oligomycin demonstrating that the AldoB-KO hepatocytes could not compensate for mitochondrial inhibition by upregulating glycolysis. Additionally, the maximal possible glycolytic rate was reduced in AldoB-KO vs WT hepatocytes. Addition of FCCP increased ECAR in hepatocytes of both genotypes, though a greater maximal rate of glycolysis was observed in WT versus AldoB-KO hepatocytes.

Next, we tested whether AldoB-KO hepatocytes metabolize long-chain fatty acids when in glucose-limited conditions to mimic the fasting state. OCR was first measured in low glucose conditions (0.5 mM) (Figure 6C). Under glucose limited-conditions differences were minimal between WT and AldoB-KO hepatocytes. Basal respiration was normal in AldoB-KO hepatocytes compared to WT and oligomycin treatment reduced OCR in both genotypes. FCCP addition increased OCR in hepatocytes of both genotypes, though to a lesser extent in AldoB-KO hepatocytes as indicated by reduced maximal respiration and mitochondrial spare capacity. CPT1 inhibition with Etomoxir slightly increased basal respiration (WT = p > 0.05 and AldoB-KO = p > 0.0001) and reduced both maximal respiration (WT = p > 0.0001, AldoB-KO = p > 0.05) and spare capacity (WT = p > 0.0001, AldoB-KO = p > 0.0001) in hepatocytes of both genotypes.

To test whether AldoB-KO hepatocytes could oxidize exogenous fatty acids in nutrient deficient conditions, palmitate was added to hepatocytes cultured in glucose-limited conditions. Basal respiration in both genotypes was not affected by palmitate addition. However, the presence of palmitate increased maximal respiration and spare capacity significantly in WT but not in AldoB-KO hepatocytes. Treatment with etomoxir reduced maximal respiration and spare capacity in WT and AldoB-KO hepatocytes incubated with palmitate suggesting that under these nutrient limited conditions, exogenous palmitate was oxidized. However, palmitate utilization was greatly compromised in AldoB-KO vs WT hepatocytes as maximal respiration and spare capacity were substantially higher in WT cells. Taken together, this data suggests that in limited glucose conditions, WT, but not AldoB-KO hepatocytes, can utilize exogenous palmitate for oxidation.

### 3.6 Fasted AldoB-KO rats have altered glucose production

As discussed above, we hypothesized that hepatic glycolytic metabolite accumulation due to impaired gluconeogenesis in fasting could promote ChREBP activation. Although the AldoB-KO rats were not hypoglycemic, fasting glucose levels were consistently lower in AldoB-KO rats (Figure 2A), consistent with data from humans with HFI where lower fasting glucose levels were reported despite normal glucose homeostasis [9]. Previous studies in mice lacking Aldob also demonstrated impaired glucose production despite normal fasting glucose levels, though fasting hepatic lipid metabolism was not assessed [37,38]. Therefore, gluconeogenesis was measured to understand whether the process was impaired in AldoB-KO rats.

**Figure 7:**
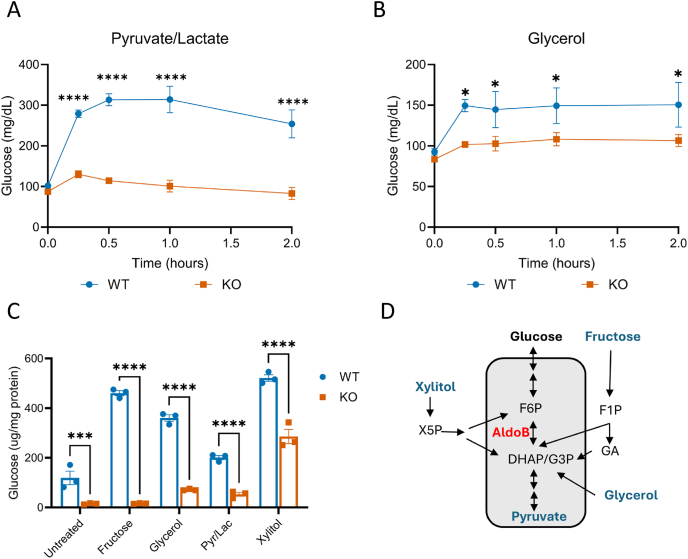
**AldoB-KO rats have impaired glucose hepatic glucose production.** (**A,B**) IP Pyruvate/Lactate Tolerance Test (P/L at a 1:10 ratio respectively) and IP Glycerol Tolerance Test in 17-week-old male WT and KO rats (n = 3–4 per group). (**C**) Glucose produced from hepatocytes incubated 1 μM glucagon for prior to incubation with 10 mM fructose, 5 mM glycerol, 1:10 (2:20 mM) pyruvate/lactate (Pyr/Lac), or 10 mM xylitol for 5 h (n = 3). (**D**) Schematic outlining how metabolites selected are integrated into glycolysis for gluconeogenesis. Statistical significance determined by 2-way Anova with Uncorrected Fisher's LSD test to compare means at each individual timepoint (A and B) and Sidak's test for multiple comparisons (**C**). ∗p < 0.05 ∗∗p < 0.01 ∗∗∗p < 0.001 ∗∗∗∗p < 0.0001. For C, specific comparisons shown and all WT treatments were p < 0.0001 (except Pyr/Lact p < 0.05) compared to WT untreated.

To assess gluconeogenesis, plasma glucose was measured in female AldoB-KO rats following administration of pyruvate/lactate (at a 10:1 M ratio) or glycerol. Injection of either pyruvate/lactate or glycerol increased plasma glucose levels significantly in female WT, but not AldoB-KO rats, confirming impaired glucose generation in the AldoB-KO rats (Figure 7A,B). To understand if the impairment was autonomous to hepatocytes, glucose production was also measured in WT and AldoB-KO hepatocytes cultured with several gluconeogenic substrates (Figure 7C). In baseline culture conditions, without the addition of gluconeogenic substrates, decreased levels of glucose were measured from AldoB-KO versus WT hepatocytes. Addition of fructose, glycerol, and pyruvate/lactate robustly stimulated glucose production in WT hepatocytes, but not in AldoB-KO hepatocytes. Xylitol was also utilized as a gluconeogenic substrate due to is conversion to xylitol-5-phosphate and further pentose phosphate metabolism allowing for a partial bypass of the aldolase reaction [39] (Figure 7D). Addition of xylitol robustly stimulated glucose production in WT hepatocytes and partially rescued it in AldoB-KO hepatocytes. Thus, reduced aldolase activity impairs glucose production in rat hepatocytes, likely augmenting hepatic glycolytic intermediates in fasted AldoB-KO rats (Figure 5D).

### 3.7 Humans with HFI are at greater risks of developing MASH

Adherence to fructose avoidance is assumed to resolve most hepatic issues in subjects with HFI [22]. Despite improvements in overall health, recent evidence indicates that people with HFI may still be at risk for hepatic steatosis and Type 2 Diabetes even after elimination of dietary fructose [26,40,41]. A meta-analysis of healthcare claims databases revealed a strong association between HFI and T2D [10]. Additionally, recent studies have shown increased liver fat and reduced ketones in fasted HFI subjects [26,40], consistent with data observed in our AldoB-KO rats. Therefore, we sought to further explore the risk of MASLD and MASH in humans with HFI.

**Figure 8:**
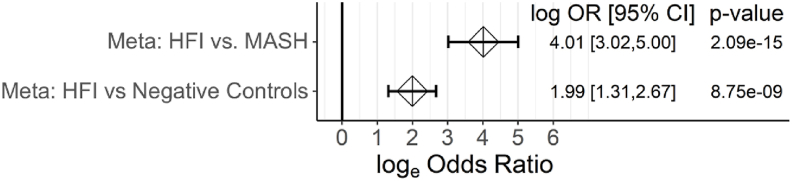
**HFI is associated with increased risk for MASH.** Forest plot of the fixed effects meta-analysis of MASH on HFI from A (log OR = 4.01, plotted at the top with 95% CI) compared to the empirical null of HFI vs. negative controls (log OR = 1.99, plotted at the bottom with 95% CI), where the latter is composed of the fixed effects meta-analysis of HFI vs. each of four negative control diseases (acute lymphoid leukemia, cervical dystonia, epilepsy, and multiple sclerosis) across the three databases, then further meta-analyzed. Meta, meta-analysis.

To understand the risk of MASLD and MASH in subjects with HFI, we conducted a meta-analysis of three healthcare claims databases focused on increased associations with MASH. MASH was chosen as the outcome of interest because of the increase in hepatic steatosis noted in the AldoB-KO rats and because of observed liver disease in people with HFI. The databases Optum® Humedica, IBM MarketScan, and IQVIA US PharMetrics, with denominators of 48,990,772, 42,363,876 and 77,779,386, respectively, were examined for HFI and MASH claims and the unadjusted odds ratio (OR) with 95% CI were subsequently calculated. In total, our cohort included 378 individuals with HFI, 71,585 individuals with MASH, and 1,070,235 with negative control diseases. All three databases showed a positive association for MASH co-occurrence with HFI (log(OR) > 2.5) (Supplemental Fig. 4). A meta-analysis of the three databases estimates the log(OR) of 4 (log(OR) = 4.01, 95% CI 3.02,5.00; p = 2.09 × 10^−15^). Previous work has shown that use of observational databases for this type of work can lead to inflated estimates for associations between diseases due to ascertainment biases [10]. To control for this, we employed the method of Desai et al., using diseases that are unrelated to MASH or HFI as negative controls to account for ascertainment bias. Similar to Desai et al., the meta-analysis across all four negative control diseases and across the three databases showed a positive association of co-occurrence between HFI and the negative controls (Figure 8) (log(OR) = 1.99; 95% CI: 1.31, 2.67, p = 8.75 × 10^−9^). We used this negative control meta-analysis to correct for the possible bias in the estimate of MASH on HFI by calculating the difference in the log odds ratios (equivalent to the log of the ratio of odds ratio, or log(ORR)) between HFI vs MASH and HFI vs. negative controls. The association of HFI vs. MASH remained significant after this adjustment (log(ORR) = 2.0, 95% CI: 0.8, 3.2, p = 0.043) supporting the hypothesis that subjects with HFI have greater risk of developing severe chronic liver disease versus subjects without HFI.

### 3.8 Inhibition of hepatic ACC prevents metabolic dysfunction in AldoB-KO rats

Our data supports the concept that loss of ALDOB activity may drive fructose-independent steatosis, hyperlipidemia, DNL and impair fatty acid oxidation upon fasting. We demonstrated that upon fasting, gluconeogenesis was impaired in AldoB-KO rats leading to glycolytic metabolite accumulation, subsequent activation of ChREBP and dysregulation of hepatic lipid metabolism. To determine the requirement of ChREBP in promoting defective fasting-induced hepatic lipid metabolism, an antisense oligonucleotide (ASO) targeting both the α and β isoforms of ChREBP previously shown to reverse diet-induced hyperlipidemia in rats [13] was utilized. Administration of the ASO decreased the mRNA expression of both isoforms in fasted WT and AldoB-KO rat livers (Supplemental Fig. 5A and B). Expression of the ChREBP target genes *Slc2a5*, *Pklr*, and *G6pc* decreased in ASO-treated rats (Supplemental Fig. 5B). Treatment with the ChREBP ASO did not significantly alter hepatic triglyceride levels in fasted AldoB-KO or WT rats (Supplemental Fig. 5C). However, the ASO prevented hypertriglyceridemia and elevations in FFA in AldoB-KO rats (Supplemental Fig. 5D and E). Additionally, a non-significant trend for increased β-OHB was observed in fasted AldoB-KO rats (Supplemental Fig. 5F). ChREBP targeting is insufficient to fully normalize hepatic lipid metabolism. Further activation of FAOx may be required to fully normalize hepatic lipid metabolism.

Next, we speculated that the increased DNL and malonyl-CoA observed in fasted AldoB-KO rats was perhaps due to increased fasting ACC activity, whose transcriptional levels were increased in AldoB-KO rat livers (Figure 4C). We reasoned that the inappropriate activation of ACC during fasting could underly the hepatic dysfunction observed in AldoB-KO rats. To test this hypothesis, fed and fasted AldoB-KO rats of both sexes were orally administered 30 mg/kg of a hepato-selective ACC1/2 inhibitor GS-0976 (Firsocostat, ACCi) [12] immediately prior to an overnight fast. A second dose was administered 12 h later, and rats were sacrificed 4 h after the last dose. Consistent with previous observations, hepatic and plasma triglycerides were increased in fasted AldoB-KO rats of both sexes compared to WT controls (Supplemental Fig. 6A and B). Treatment with hepato-selective ACCi reduced hepatic triglycerides in AldoB-KO rats in both sexes when compared to vehicle treated AldoB-KO rats. No difference was observed in plasma triglyceride levels between ACCi and vehicle treated male and female AldoB-KO rats. Levels of β-OHB also trended higher in AldoB-KO male (Vehicle = 105.1 +/− 5.7 SEM, and ACCi = 161.2 +/− 19.4 SEM) and female (Vehicle = 141 +/− 38.4 SEM and ACCi 173.3 +/− 19.0 SEM) rats with ACCi treatment suggesting a slight elevation in fat oxidation in AldoB-KO rats (Supplemental Fig. 6C). However, levels of β-OHB were still significantly below WT levels. Changes β-OHB levels were not observed in WT fasted rats using this treatment paradigm.

**Figure 9:**
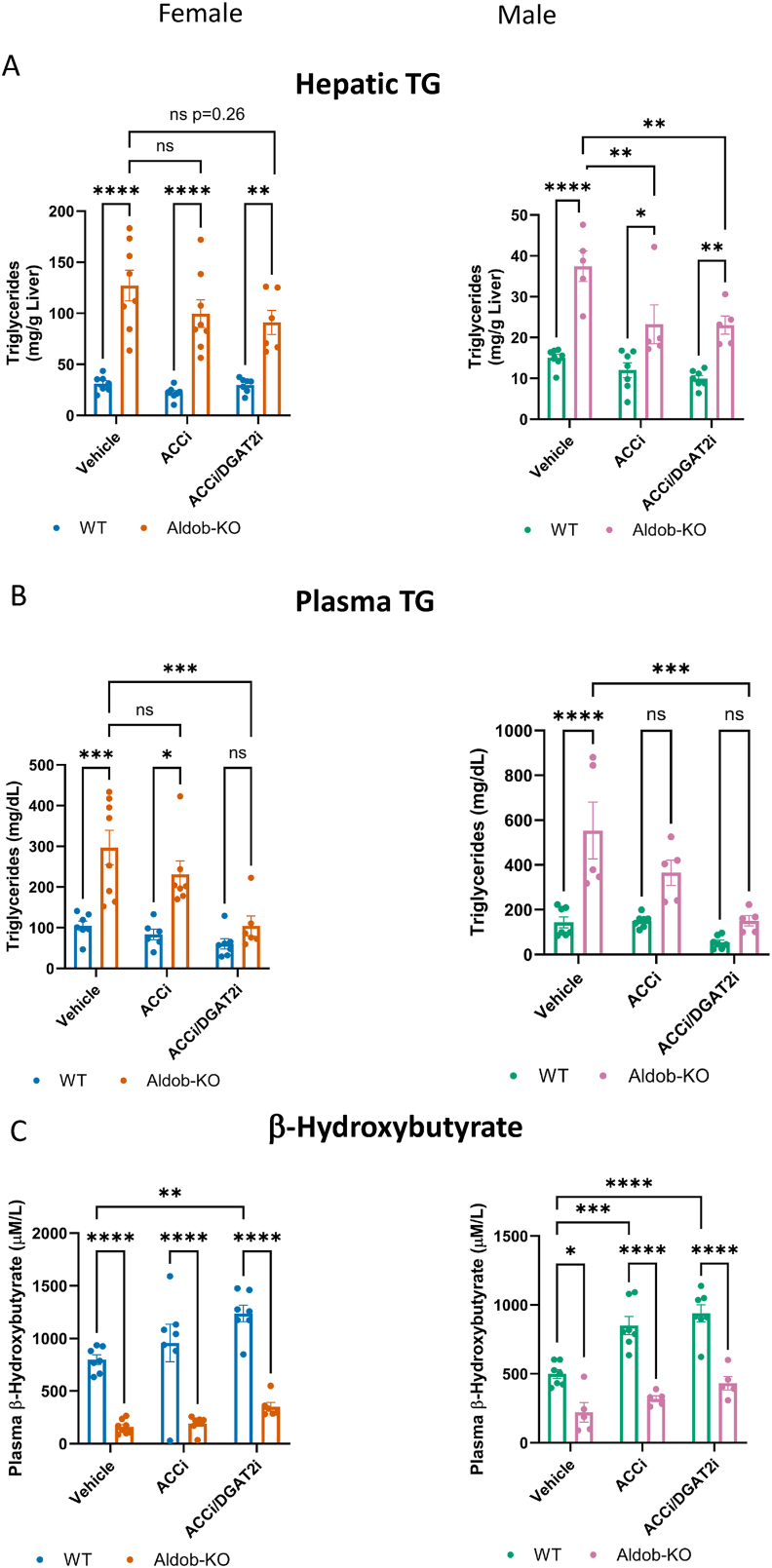
**Inhibition of ACC and DGAT2 reduces hepatic and circulating triglycerides in AldoB null rats.** (**A**) Hepatic and (**B**) plasma triglycerides in 15–16-week-old female and male WT and AldoB-KO rats treated with and ACC inhibitor or ACC inhibitor plus DGAT2 inhibitor. (**C**) Levels of β-OHB in rats treated with the inhibitors. Statistical significance determined by 2-way Anova with Sidak's test for multiple comparisons. ∗p < 0.05 ∗∗p < 0.01 ∗∗∗p < 0.001 ∗∗∗∗p < 0.0001. ns = non-significant.

Studies in humans and rodents have shown that ACC inhibitors increase plasma triglycerides due to activation of SREBP1c [42,43]. One strategy employed to mitigate the activation of SREBP with ACCi has been to co-administer a Diacylglycerol transferase 2 inhibitor (PF-06427878) (DGAT2i) [42]. Inhibition of DGAT2 suppresses SREBP activation in both genetic and pharmacologic models [42,[44], [45], [46]]. Therefore, we tested whether greater therapeutic benefit would be achieved through co-administration of an ACCi/DGAT2i combination. As before, rats were treated with vehicle, 30 mg/kg of ACCi or 30 mg/kg of ACCi and DGAT2i each, 12 h apart with the first dose coinciding with the start of the fast. Rats were sacrificed 4 h post final dose. In female AldoB-KO rats, hepatic triglycerides trended partially lower with both ACCi, and the ACCi/DGAT2i treatment (Figure 9A). In male rats ACCi and ACCi/DGAT2i significantly reduced hepatic triglycerides, though as observed in female rats, no further lowering was observed with the combination when compared to ACCi alone. Interestingly, the administration of the ACCi/DGAT2i combination did enhance reductions in plasma triglycerides when compared to ACCi alone, fully normalizing the plasma triglycerides in AldoB-KO rats to WT levels in both sexes (Figure 9B). In this study β-OHB levels increased in male and female rats of both genotypes with ACCi and even further with ACCi/DGAT2i treatment demonstrating target engagement, though the effect was only significant in WT rats (Figure 9C). Thus, co-administration of ACCi and DGAT2i reduced hepatic triglycerides and fully normalized circulating triglycerides in AldoB-KO rats.

Finally, we could not exclude the possibility that endogenous fructose production via polyol metabolism elevated triglycerides in AldoB-KO rats as observed in AldoB-KO mice provided glucose sweetened water [47]. We also reasoned that the trace amounts of dietary fructose in diet D16121902 could not be causative since steatosis and hyperlipidemia was primarily observed in fasted and not fed rats. However, to rule out either polyol fructose generation or trace dietary fructose, we tested the effect of PF-06835919, a potent fructokinase inhibitor (KHKi) [23,48] in fed and fasted AldoB-KO rats. As observed previously, F1P levels were higher in fed and fasted AldoB-KO compared to WT rats (Supplemental Fig. 7A). Treatment with the KHKi significantly lowered F1P in AldoB-KO rats in both nutritional states, though F1P levels remained above WT levels likely due to the slow turnover of this metabolite pool without sufficient ALDOB activity. Hepatic triglycerides, circulating triglycerides and free fatty acids were all elevated while the fatty acid oxidation marker β-OHB was suppressed consistent with our previous results (Supplemental Fig. 7B–E) and treatment with KHKi did not normalize any of these endpoints. Hepatic expression of ChREBP target genes remained elevated in AldoB-KO rats treated with KHKi (Supplemental Fig. 7F). Together this data suggests that fructose independent metabolic dysfunction exists with lack of ALDOB activity, and novel therapies such as the ACCi/DGAT2i combination may offer a therapeutic benefit to subjects with HFI.

## 4 Discussion

Here we sought to understand if fructose-independent metabolic dysfunction exists in HFI and underlies the metabolic risk for liver steatosis and metabolic disease. As noted, fructose abstinence has been suggested to resolve and prevent hepatic pathologies, allowing people with HFI to live a disease-free life [22]. Despite vigilantly fructose avoidance, liver pathologies exist years after HFI diagnosis and Type 2 diabetes risk remains in people with HFI [10,26,40]. Our results explain why people with HFI have increased metabolic risk despite fructose avoidance.

AldoB-KO rats faithfully recapitulated human HFI, serving as an appropriate model for studying fructose independent metabolism in HFI. Consistent with observations in humans with HFI, fructose administration to AldoB-KO rats reduced glycemia likely due to both impaired hepatic glucose output from F1P accumulation, a known allosteric activator of glucokinase and inhibitor of hepatic glucose production [49,50], and from impaired conversion of fructose to glucose [23]. Exposure of AldoB-KO neonates to low amounts of dietary fructose reduced body weights and caused hepatic fibrosis, both reported in pediatric patients with HFI exposed to fructose [9].

Metabolic phenotyping revealed that fed AldoB-KO rats were healthy. Upon fasting, AldoB-KO rats developed hepatic steatosis, hyperlipidemia, and hypoketonemia due to elevated DNL and reduced fatty acid oxidation. The liver is a dynamic organ and shifts between catabolic and anabolic processes quickly depending on nutritional status. In the fed state the liver largely synthesizes lipids through DNL and stores glucose as glycogen. When fasting, DNL ceases in favor of fatty acid oxidation and glucose production. The activation of DNL in fasted AldoB-KO rats was determined using D_2_O labeling to label newly synthesized palmitate, a common in vivo technique for assessing rates of DNL in mammals [[51], [52], [53]]. Using this technique, we demonstrated that lipogenesis was elevated in AldoB-KO rats despite fasting and not suppressed as in WT rats. Surprisingly, fasted AldoB-KO rats also did not oxidize fatty acids as β-hydroxybutyric acid levels remained low compared to WT rats. This finding was confirmed in isolated hepatocytes which also failed to oxidize fatty acids in nutritionally limited media. As discussed above, plasticity to switch between lipogenesis and fat oxidation is important for normal hepatic physiologic responses to nutrient status. The fact that the metabolic perturbations were only evident upon fasting suggests that ALDOB is dispensable for hepatic metabolism in the fed state if fructose is absent. However, even without dietary fructose, loss of ALDOB prevents hepatocytes from shifting towards normal hepatic fasting metabolism. Thus, while ALDOB is dispensable for fed liver metabolism, it is essential for fasting.

In agreement with this notion, we observed impaired gluconeogenesis in AldoB-KO rats and their hepatocytes. Interestingly, despite having impaired gluconeogenesis, AldoB-KO rats never developed severe hypoglycemic during a 16–18 h fast suggesting that other sources of glucose production compensate. This result is consistent with observations from humans with HFI where hypoglycemia was not observed even after an overnight fast [54,55]. While lack of hypoglycemia suggests that gluconeogenesis is intact in people with HFI, to our knowledge, quantitative measurements of hepatic glucose production have not been performed in subjects with HFI. In mice lacking *Aldob*, impaired glucose production from pyruvate was reported despite normal fasting glucose [38] and confirmed in our studies with AldoB-KO rats administered Pyruvate/Lactate or glycerol. These substrates also failed to increase glucose production directly in AldoB-KO hepatocytes confirming the hepatocyte impairment. Importantly, xylitol, a metabolite that partially bypasses the aldolase reaction somewhat restored glucose production in AldoB-KO hepatocytes. While it is known that glucose can be produced from the kidneys and intestines [56,57], the contribution of these organs to gluconeogenesis in humans and AldoB-KO rats remains unknown. Understanding how normoglycemia is maintained with loss of *Aldob* is an interesting question for future studies.

The fact that hepatocytes also contain a second Aldolase enzyme, Aldolase A, which is fully capable of mediating the glycolytic aldolase step raises the question of why this enzyme does not compensate for lack of ALDOB in the fasted state. Like ALDOB, ALDOA also facilitates the aldolase reaction in glycolysis bi-directionally [4]. However, ALDOA is expressed at ∼1/20th the level of ALDOB in rat livers [58]. Additionally, while ALDOA is catalytically more efficient at the cleavage of F1,6P, ALDOB is more efficient synthesizing F1,6P from trioses [4]. ALDOA is likely incapable of fully compensating for the loss of ALDOB, especially upon fasting, explaining why hepatic glucose production is inhibited in rats lack *Aldob*.

Through our detailed metabolomic studies, we confirmed the impairment in metabolism at the aldolase reaction and the inability of *Aldoa* to compensate for loss of ALDOB activity. In fed AldoB-KO livers very few metabolites were different between WT and AldoB-KO rats. Primarily, G6P, F6P and the ALDOB substrates F1P and F1,6P were elevated, likely due to impaired catabolism of F1,6P and F1P. Upon fasting, many hepatic glycolytic metabolites, especially three- and two-carbon intermediates, accumulated in AldoB-KO livers, likely due to impaired gluconeogenesis discussed above. F1P, an allosteric activator of glucokinase [49] remained elevated during fasting. Thus, F1P-induced glucokinase activation may partly suppress hepatic glucose production in AldoB-KO rats. Consistent with the deficiency in FAOx and increase in DNL, acetyl-CoA levels were reduced while malonyl-CoA levels were increased. Fasting induced multiple metabolic changes likely due to impairment at the aldolase reaction in glycolysis.

The observed increase in hepatic metabolites during fasting in AldoB-KO rats likely drives the dysregulation of lipid metabolism through the activation of ChREBP. Lipogenesis is controlled in mammals through the coordinated regulation of the transcription factors ChREBP and SREBP [30,32,59]. Upon fasting, activity of both transcription factors normally decreases. In fasted AldoB-KO livers, ChREBP, but not SREBP, target gene expression was increased, suggesting hyperactivation of the transcription factor. Activation of ChREBP is important for lipogenesis as the absence of ChREBPα and ChREBPβ blunts hepatic lipid synthesis and overexpression of ChREBPα in mice causes hepatic steatosis [28,31,60]. Previous studies proposed that sugar phosphates such as G6P, F2,6P or xylulose-5 phosphate activate ChREBP [[61], [62], [63]]. Interestingly, upon fasting hepatic G6P decreased and was not different with regards to AldoB-KO and WT rats suggesting another metabolite might be responsible for ChREBP activation in our studies. Accordingly, xylulose-5 phosphate levels were found to be 9.2-fold greater in fasted AldoB-KO rat livers compared to WT (Supplemental Table 5). Unfortunately, we were unable to accurately detect F2,6P with our current analytical method and thus we cannot ascertain whether this metabolite may be responsible for the ChREBP activation observed upon fasting. More recently, a publication suggested that altered redox state and elevated levels of trioses such as DHAP and glyceraldehyde-3 phosphate are responsible for ChREBP activation [64]. Though we unable to measure glyceraldehyde-3 phosphate with our method, DHAP levels were detectable and found to decrease upon fasting in WT livers but not in AldoB-KO livers where it remained over 6.5-fold higher vs WT. Regardless of which metabolite(s) may be activating ChREBP, our data is consistent with the concept that the buildup of glycolytic metabolites activates ChREBP, which in turn may be responsible for augmenting DNL and blocking FAOx in fasted AldoB-KO rats.

These findings are overall consistent with studies in humans with HFI. As mentioned above, persistent steatosis and fibrosis has been reported in subjects with HFI years after cessation of fructose consumption [9]. Reduced levels of β-hydroxybutyric acid and elevated hepatic triglycerides have also been reported in fasted individuals with HFI relative to matched control subjects [26]. In another study Di Dato et al. showed that despite consuming approximately 10X lower fructose than the recommended 1.5 g/day limit, 37.5% subjects with HFI had elevated transaminases and 93.8% had steatosis evaluated on average 10.3 years after initial diagnosis [65]. Furthermore, use of electronic medical health databases to examine a broader population of subjects with HFI demonstrated an increased incidence of Type-2 diabetes in people with HFI compared to controls [10]. Following up on this work, we utilized electronic medical health records and discovered that MASH diagnosis was increased about 2-fold when corrected for non-related diseases, further demonstrating that subjects with HFI may have inherent metabolic dysfunction that is likely fructose-independent.

There are several limitations with our studies that can be addressed in future work. In our work we sought to identify potential therapies to reduce steatosis and hyperlipidemia in AldoB-KO rats upon fasting. While neither ChREBP nor ACC inhibition alone fully resolved metabolic abnormalities observed upon fasting, each strategy resolved some aspects. Notably, combining ACCi with DGAT2i improved both hepatic and circulating triglycerides in fasted AldoB-KO rats. However, steatosis and FAOx were not fully normalized. How FAOx is blocked in AldoB-KO rats also remains unknown. Though activation of ChREBP has been suggested as a mechanism for suppressing FAOx [28] our data does not fully support this hypothesis as ChREBP inhibition only slightly elevated FAOx.

How normal glycemia is maintained in fasted AldoB-KO rats despite limited hepatic glucose production remains unknown. As mentioned earlier, Aldolase B is also highly expressed in the kidney and small intestines, organs important for glucose and lipid metabolism [56,57]. Although glucose production was impaired in AldoB-KO rat hepatocytes, we did not assess whether loss of ALDOB also affected gluconeogenesis renal proximal tubule or intestinal epithelial cells. Future studies will be needed to understand if the kidney or the intestines compensate for loss of hepatic gluconeogenesis with ALDOB.

Whether impaired gluconeogenesis with the loss of ALDOB impairs FAOx will also need to be resolved in future studies. Gluconeogenesis and FAOx are indeed closely linked as mice with liver-specific PEPCK KO are phenotypically similar to the AldoB-KO rats. Normal glycemia, fasting steatosis, hypertriglyceridemia and hypoketonemia, as observed in our studies with AldoB-KO rats, have been reported in PEPCK KO mice [66,67]. If impaired gluconeogenesis drives the hepatic phenotype observed in AldoB-KO rats, gene replacement may be necessary to restore normal hepatic metabolism.

How DNL remains elevated in fasted AldoB-KO rats remains unknown. Impaired gluconeogenesis and glycolytic metabolite accumulation below the aldolase reaction could theoretically increase flux of potential gluconeogenic substrates into the TCA cycle, providing citrate and other lipogenic precursors. This scenario would cause metabolites such as glycerol and lactate to be shunted towards lipogenesis rather than gluconeogenesis. While these metabolites could serve as lipogenic substrates in AldoB-KO hepatocytes, other metabolites may also contribute. Recently Liao et al. demonstrated that amino acids, not glucose, are the major carbon source for DNL in hepatocytes [68]. Therefore, AldoB-KO hepatocytes may also augment lipogenesis by increasing utilization of TCA intermediates derived from amino acids. Future studies using metabolite tracers are needed to clarify the question of which metabolic pathways are driving DNL in AldoB-KO rats.

Finally, as observed with rodent diets, the complete elimination of dietary fructose in humans is virtually impossible as trace amounts likely are present as additives or contaminants for processed foods. Nonetheless, we do not believe that the trace amounts of fructose in the diet were responsible for the phenotype observed. Under fed conditions differences in body weight, liver triglycerides, circulating metabolites and overall metabolic health were not detected. The phenotype reported was exclusive to fasting, long after cessation of dietary fructose absorption and metabolism. It is plausible that fasting conversion of glucose to fructose by means of polyol metabolism could explain the phenotype observed in AldoB-KO rats. However, no benefit was observed with KHK inhibition further supporting the concept that the metabolic dysfunction observed upon fasting in AldoB-KO rats was independent of fructose metabolism.

**Figure 10:**
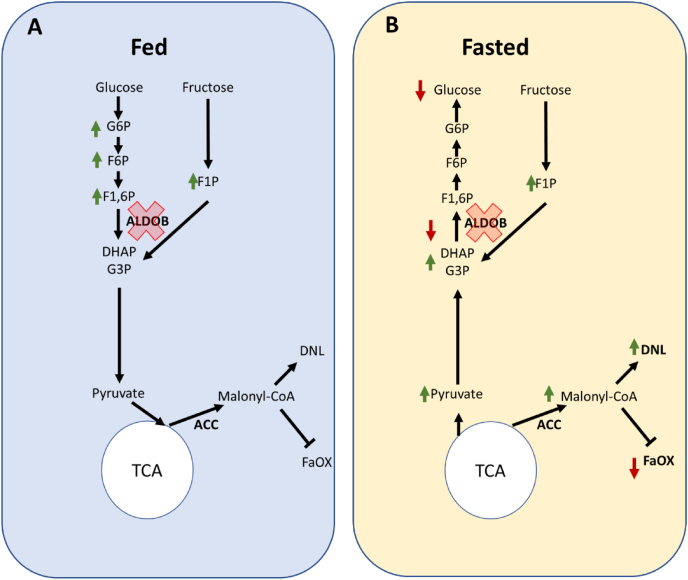
**Loss of Aldolase B causes impaired glycolytic metabolism in the fasted state**. Model showing how loss of Aldolase B impacts fructose and glycolytic metabolism is the (**A**) fed and (**B**) fasted state. Green and red arrows indicate metabolites and processes increased or decreased. Image made in Microsoft PowerPoint.

In summary, this work suggests the following model. In the fed state, hepatic metabolism is largely normal outside of fructose metabolism (Figure 10A). Upon fasting, gluconeogenesis is impaired, leading to an increase in glycolytic metabolites which in turn activate ChREBP (Figure 10B). Both the activation of ChREBP and accumulation of metabolites like Malonyl-CoA promote steatosis and hyperlipidemia through the activation of DNL and suppression of FAOx. Future studies will be needed to understand the translation of these findings to humans. Our work does suggest that some of the metabolic dysfunction, including steatosis and hyperlipidemia, reported in individuals with HFI could be independent of dietary fructose. Therapies may be needed to address the increased risk of MASH and Type-2 diabetes reported in people with HFI.

## Study approval

Experiments were approved by the Institutional Animal Care and Use Committee of Pfizer Inc. and conducted in accordance with local, state, federal guidelines and the Guide for the Care and Use of Laboratory Animals from the National Institutes of Health. All data sources utilized were fully compliant with the U.S. privacy laws and federal regulations such as the Health Insurance Portability and Accountability Act (HIPAA). Patient informed consent was not required as the databases were de-identified.

## CRediT authorship contribution statement

**Melissa A. Fulham:** Writing – review & editing, Writing – original draft, Supervision, Methodology, Investigation, Formal analysis, Data curation, Conceptualization. **John D. Griffin:** Investigation, Formal analysis. **Sylvie Perez:** Writing – original draft, Investigation, Data curation. **Zhongyuan Sun:** Methodology, Investigation, Formal analysis, Data curation. **Natalie A. Daurio:** Investigation, Data curation. **Gang Xing:** Methodology, Investigation. **Michelle F. Clasquin:** Methodology, Formal analysis, Conceptualization. **Melissa R. Miller:** Writing – original draft, Methodology, Formal analysis, Data curation. **Craig L. Hyde:** Writing – original draft, Methodology, Investigation, Formal analysis, Conceptualization. **Scott P. Kelly:** Methodology, Formal analysis, Data curation, Conceptualization. **Magalie Boucher:** Formal analysis, Data curation. **Rachel Poskanzer:** Investigation, Formal analysis, Data curation. **Ramya Gamini:** Writing – original draft, Methodology, Formal analysis, Data curation, Conceptualization. **Evanthia Pashos:** Conceptualization. **Ying Zhang:** Investigation, Formal analysis, Data curation. **Elaine Kuang:** Investigation, Formal analysis, Data curation. **Josh Fienman:** Investigation, Formal analysis, Data curation. **Kendra K. Bence:** Writing – review & editing, Formal analysis, Conceptualization. **Gregory J. Tesz:** Writing – review & editing, Writing – original draft, Supervision, Formal analysis, Conceptualization.

## Declaration of competing interest

The authors declare the following financial interests/personal relationships which may be considered as potential competing interests: Gregory J. Tesz reports a relationship with Pfizer Inc that includes: employment and equity or stocks. Melissa A. Fulham reports a relationship with Pfizer Inc that includes: employment. Sylvie Perez reports a relationship with Pfizer Inc that includes: employment. John D. Griffin reports a relationship with Pfizer Inc that includes: employment and equity or stocks. Zhongyuan Sun reports a relationship with Pfizer Inc that includes: employment and equity or stocks. Natalie A. Daurio reports a relationship with Pfizer Inc that includes: employment and equity or stocks. Gang Xing reports a relationship with Pfizer Inc that includes: employment and equity or stocks. Michelle F. Clasquin reports a relationship with Pfizer Inc that includes: employment and equity or stocks. Melissa R. Miller reports a relationship with Pfizer Inc that includes: employment and equity or stocks. Craig L. Hyde reports a relationship with Pfizer Inc that includes: employment and equity or stocks. Scott P. Kelly reports a relationship with Pfizer Inc that includes: employment and equity or stocks. Magalie Boucher reports a relationship with Pfizer Inc that includes: employment. Rachel Poskanzer reports a relationship with Pfizer Inc that includes: employment. Ramya Gamini reports a relationship with Pfizer Inc that includes: employment. Evanthia Pashos reports a relationship with Pfizer Inc that includes: employment and equity or stocks. Ying Zhang reports a relationship with Pfizer Inc that includes: employment and equity or stocks. Elaine Kuang reports a relationship with Pfizer Inc that includes: employment. Josh Fienman reports a relationship with Pfizer Inc that includes: employment and equity or stocks. Kendra K. Bence reports a relationship with Pfizer Inc that includes: employment and equity or stocks. If there are other authors, they declare that they have no known competing financial interests or personal relationships that could have appeared to influence the work reported in this paper.

## Data Availability

Data will be made available on request.

## Appendix A Supplementary data

Supplementary Figure 1 **AldoB-KO rats fed diet D16121902 (0.02% fructose) do not lose weight or have elevated hepatic triglycerides.** (**A**) Body weights of 13-15-week-old WT and KO rats weaned onto the 0.02% fructose diet (D16121902). (**B**) Biochemical measurement of hepatic triglyceride content in 20–21-week-old WT and AldoB-KO male and female rats fed D16121902. H&E histological stain of hepatic samples from 15-week-old male and female WT and AldoB-KO rats fed D16121902. Statistical significance determined by one-way ANOVA with Tukey's multiple comparisons test. ∗p < 0.05 ∗∗p < 0.01 ∗∗∗p < 0.001 ∗∗∗∗p < 0.0001. Supplementary Figure 2**Increased expression of hepatic ChREBP target gene expression in fasted AldoB-KO rats.** Targeted qrtPCR expression analysis of hepatic gene targets of ChREBP (**A**), ChREBP/SREBP (**B**), SREBP target genes (**C**), and PPARα target genes (**D**) of 23-32-week-old fed and fasted, male, WT and KO rats, normalized to fed WT controls from two different studies combined. Two-way ANOVA with Tukey's multiple comparisons test was used to calculate statistical significance. ∗p < 0.05 ∗∗p < 0.01 ∗∗∗p < 0.001 ∗∗∗∗p < 0.0001 vs fed; #p < 0.05 ##p < 0.01 ###p < 0.001 ####p < 0.0001 vs WT. Supplementary Figure 3**Fructose metabolites accumulate in fed AldoB-KO livers.** (**A**) Volcano plot showing metabolites that are significantly different (>1.5 fold and p value < 0.05) between fed WT and AldoB-KO livers from 11-week-old rats. Supplementary Figure 4**Association of HFI with MASH as analyzed from individual electronic health record data bases.** Forest plot of three individual databases of NASH against HFI with the fixed-effects meta-analysis of the three DB under the dotted line. The vertical line represents the theoretical null of 1. Supplementary Figure 5**ASOs targeting ChREBP partially normalize metabolism in AldoB-KO rats.** (**A-G**). 19-21-week-old female WT and KO rats were treated with the ChREBP or control ASO prior to an overnight fast. (**A-C**) Hepatic gene expression analysis of ChREBP target genes (**A**) and ChREBP target genes (**B**) of AldoB-KO and WT rats treated with control or ChREBP targeting ASO. Fasted hepatic triglycerides (**C**), plasma triglycerides (**D**), plasma FFA (**E**) and plasma β-OHB (**F**) of ChREBP ASO- or control ASO-treated rats. Two-way ANOVA with Tukey's multiple comparisons test was used to calculate statistical significance. ∗p < 0.05 ∗∗p < 0.01 ∗∗∗p < 0.001 ∗∗∗∗p < 0.0001 vs WT; #p < 0.05 ##p < 0.01 ###p < 0.001 ####p < 0.0001 vs ChREBP ASO. (H–P) ∗p < 0.05 ∗∗p < 0.01 ∗∗∗p < 0.001 ∗∗∗∗p < 0.0001 vs fed; #p < 0.05 ##p < 0.01 ###p < 0.001 ####p < 0.0001 vs WT. Supplementary Figure 6**ACC inhibition reduces hepatic triglycerides in fasted AldoB-KO rats.** (**A**) Hepatic and (**B**) plasma triglycerides in 12–16-week-old female and male WT and AldoB-KO rats treated with and ACC inhibitor. (**C**) Levels of β-OHB in rats treated with the inhibitors. Statistical significance determined by 2-way Anova with Sidak's test for multiple comparisons. ∗p < 0.05 ∗∗p < 0.01 ∗∗∗p < 0.001 ∗∗∗∗p < 0.0001. ns = non-significant. Supplementary Figure 7**KHK inhibition does not reverse fasting metabolic dysfunction in AldoB-KO rats.** Hepatic F1P concentration (**A**) and triglyceride levels (**B**) in 23-24-week-old, female, fed and fasted WT and KO rats treated with vehicle or KHK inhibitor (KHKi). (**C**) Plasma triglycerides and (**D**) FFA in fed and fasted WT and AldoB-KO rats treated with vehicle or KHKi. (**E**) Fasted plasma β-OHB in fasted WT and AldoB-KO rats treated with vehicle or KHKi. (**F**) Expression of hepatic ChREBP target genes in fasted and fed WT and AldoB-KO rats treated with vehicle or KHKI. Statistical significance determined by 2-way Anova with Sidak's test for multiple comparisons. ∗p < 0.05 ∗∗p < 0.01 ∗∗∗p < 0.001 ∗∗∗∗p < 0.0001 vs fed; #p < 0.05 ##p < 0.01 ###p < 0.001 ####p < 0.0001 vs WT; +p < 0.05 ++p < 0.01 +++p < 0.001 ++++p < 0.0001 vs vehicle. Supplementary Table 1Histological score of kidneys from WT or AldoB-KO rats fed diet 2920X or D16121902. Supplementary Table 2Changes in mRNA expression of annotated PPAR signaling pathway genes (hsa03320). Supplementary Table 3Changes in mRNA expression of annotate Bile secretion pathway genes (hsa04976). Supplementary Table 4Significantly altered metabolites in fed AldoB-KO livers compared to WT. Supplementary Table 5Significantly altered metabolites in fasted AldoB-KO livers compared to WT. Supplementary Figure 1
