# Supplementary material for: Impaired hepatic metabolism in Hereditary Fructose Intolerance confers fructose-independent risk for steatosis and hypertriglyceridemia
Source: Mol Metab. 2025 Dec 19;104:102310. doi: 10.1016/j.molmet.2025.102310 (PMC12853781; doi:10.1016/j.molmet.2025.102310)
Supplement: Supplementary Figure 1 — AldoB-KO rats fed diet D16121902 (0.02% fructose) do not lose weight or have elevated hepatic triglycerides. (A) Body weights of 13-15-week-old WT and KO rats weaned onto the 0.02% fructose diet (D16121902). (B) Biochemical measurement of hepatic triglyceride content in 20–21-week-old WT and AldoB-KO male and female rats fed D16121902. H&E histological stain of hepatic samples from 15-week-old male and female WT and AldoB-KO rats fed D16121902. Statistical significance determined by one-way ANOVA with Tukey's multiple comparisons test. ∗p < 0.05 ∗∗p < 0.01 ∗∗∗p < 0.001 ∗∗∗∗p < 0.0001. Supplementary Figure 2Increased expression of hepatic ChREBP target gene expression in fasted AldoB-KO rats. Targeted qrtPCR expression analysis of hepatic gene targets of ChREBP (A), ChREBP/SREBP (B), SREBP target genes (C), and PPARα target genes (D) of 23-32-week-old fed and fasted, male, WT and KO rats, normalized to fed WT controls from two different studies combined. Two-way ANOVA with Tukey's multiple comparisons test was used to calculate statistical significance. ∗p < 0.05 ∗∗p < 0.01 ∗∗∗p < 0.001 ∗∗∗∗p < 0.0001 vs fed; #p < 0.05 ##p < 0.01 ###p < 0.001 ####p < 0.0001 vs WT. Supplementary Figure 3Fructose metabolites accumulate in fed AldoB-KO livers. (A) Volcano plot showing metabolites that are significantly different (>1.5 fold and p value < 0.05) between fed WT and AldoB-KO livers from 11-week-old rats. Supplementary Figure 4Association of HFI with MASH as analyzed from individual electronic health record data bases. Forest plot of three individual databases of NASH against HFI with the fixed-effects meta-analysis of the three DB under the dotted line. The vertical line represents the theoretical null of 1. Supplementary Figure 5ASOs targeting ChREBP partially normalize metabolism in AldoB-KO rats. (A-G). 19-21-week-old female WT and KO rats were treated with the ChREBP or control ASO prior to an overnight fast. (A-C) Hepatic gene expression analysis of ChREBP target genes [file mmc1.pdf]

Supplemental figure 1

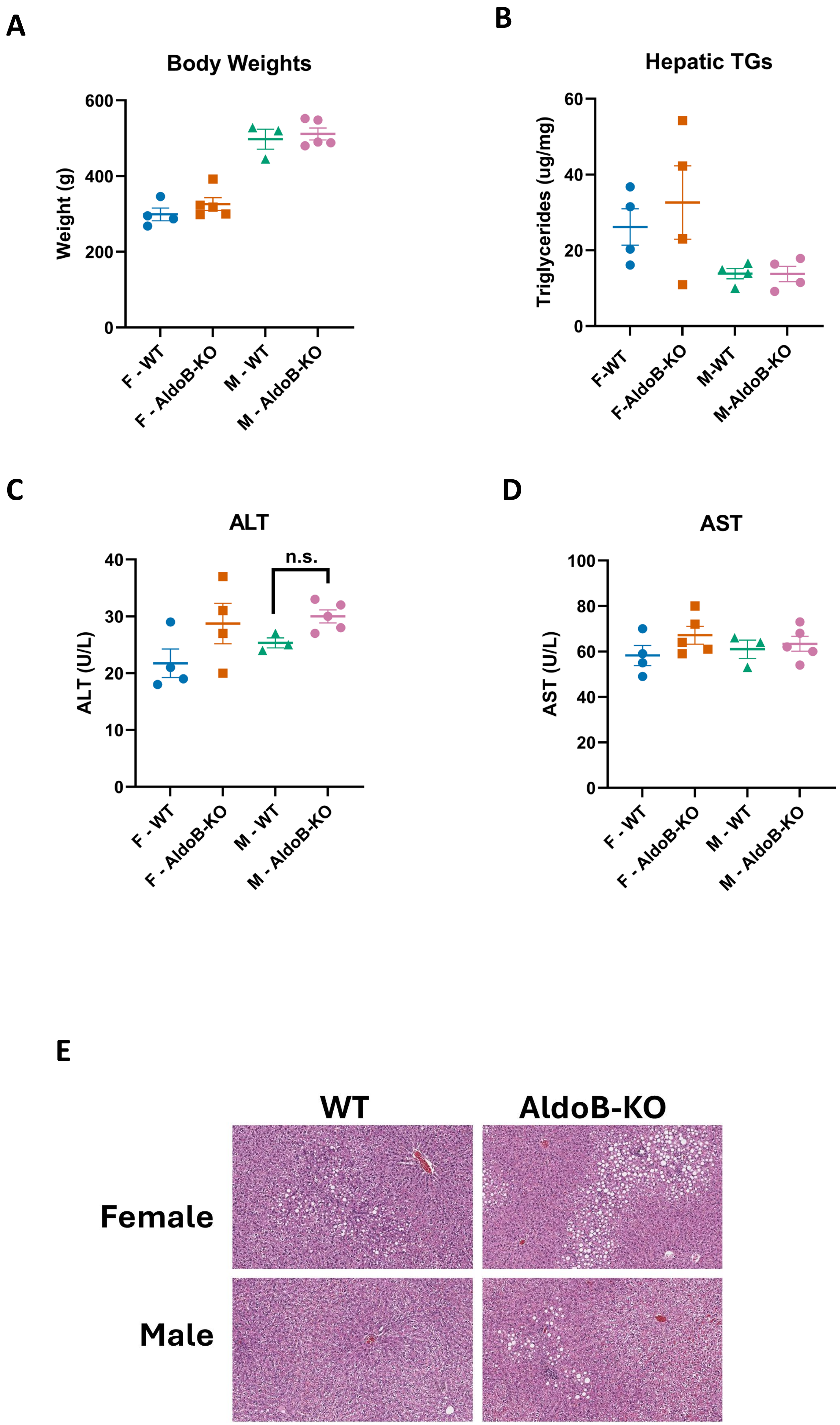

# Supplemental figure 2

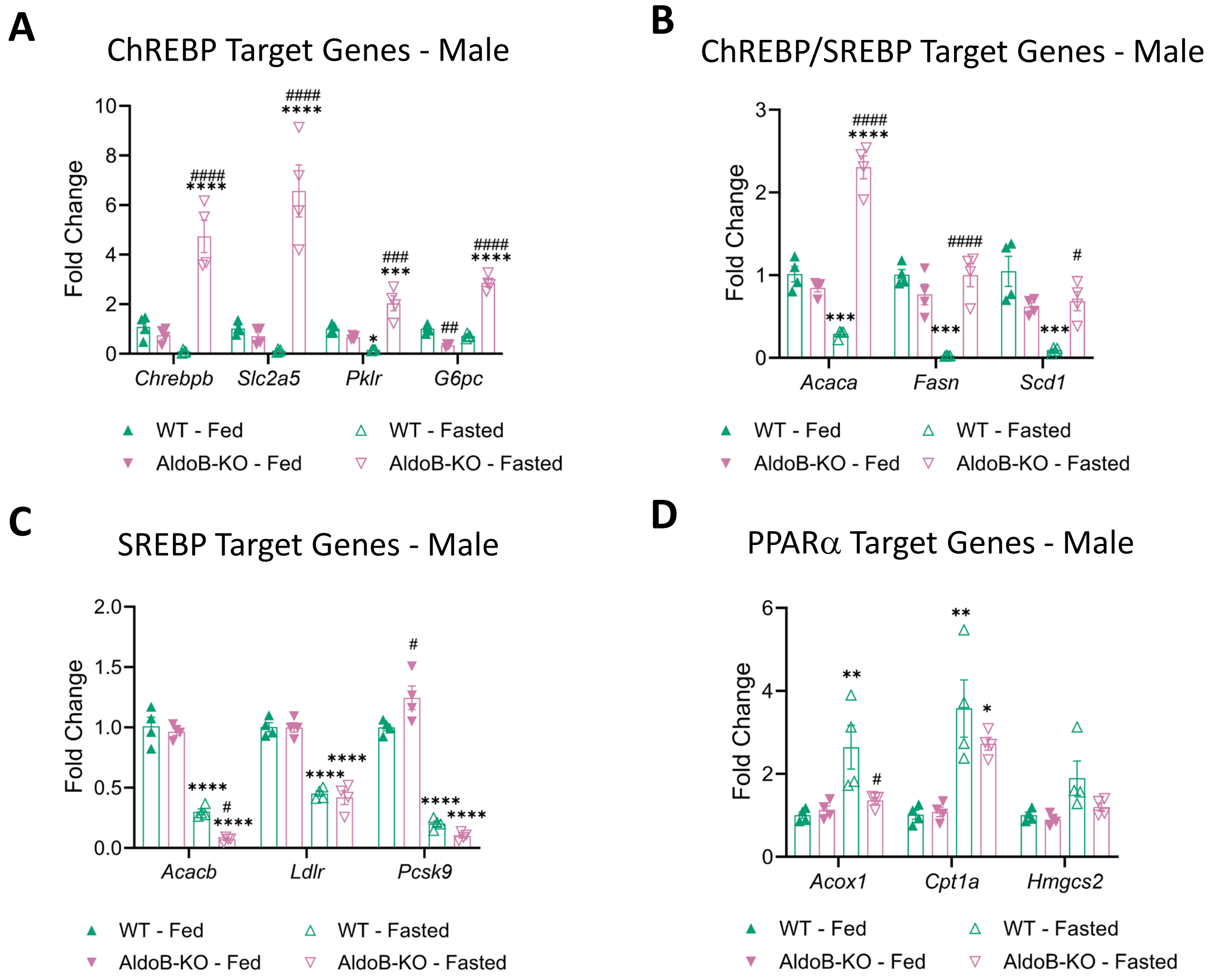

# Supplemental Figure 3

A

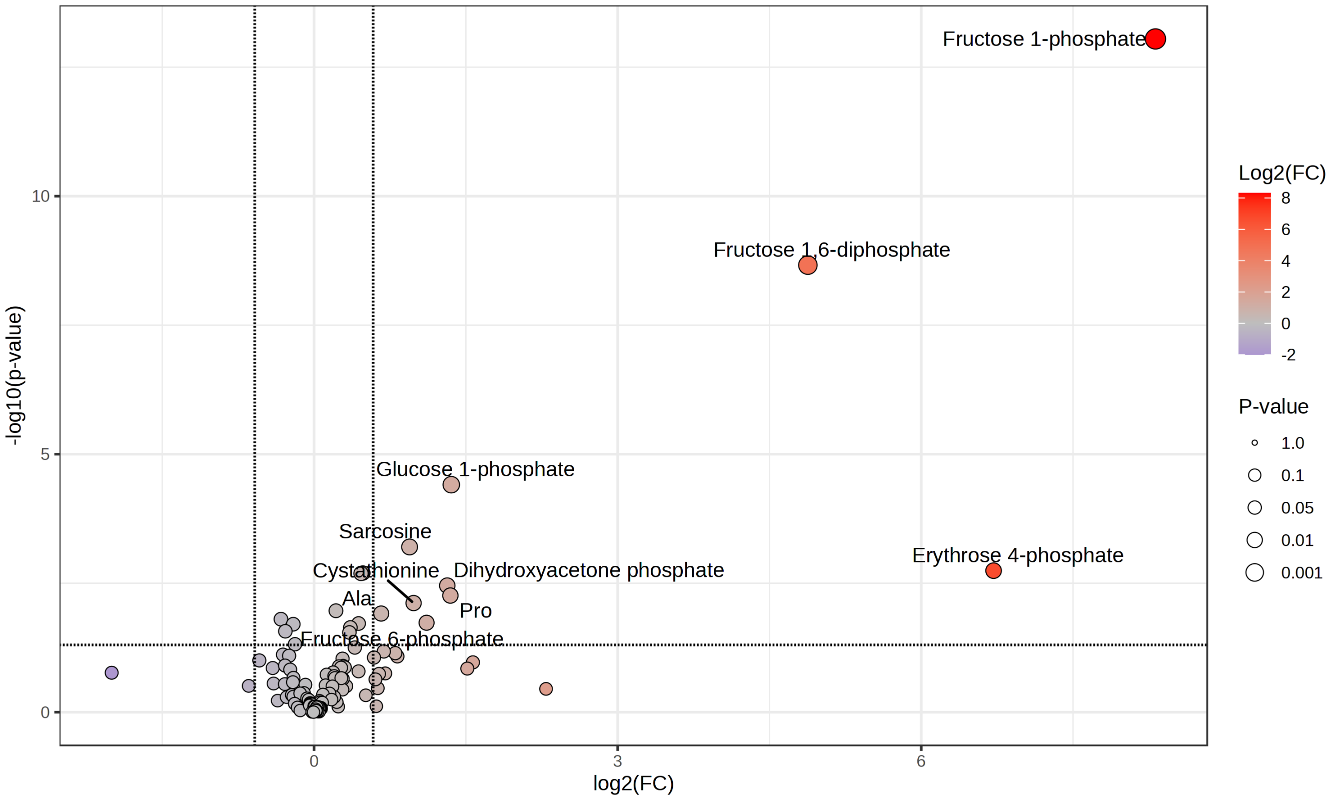

# Supplemental Figure 4

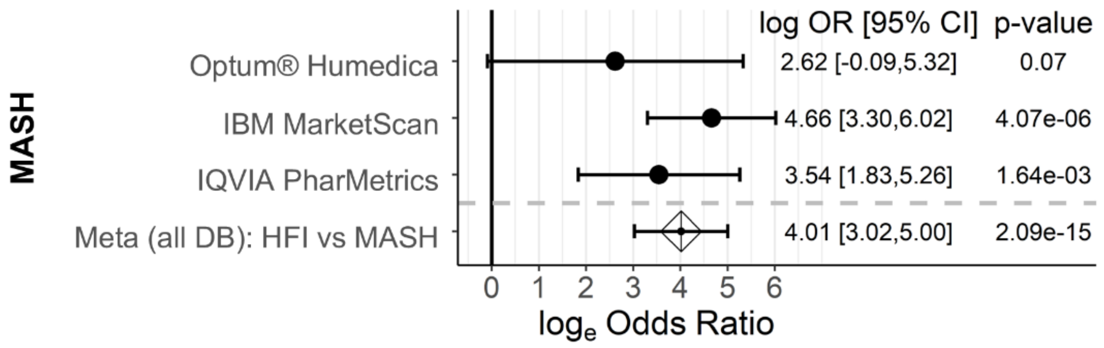

Supplemental Figure 5

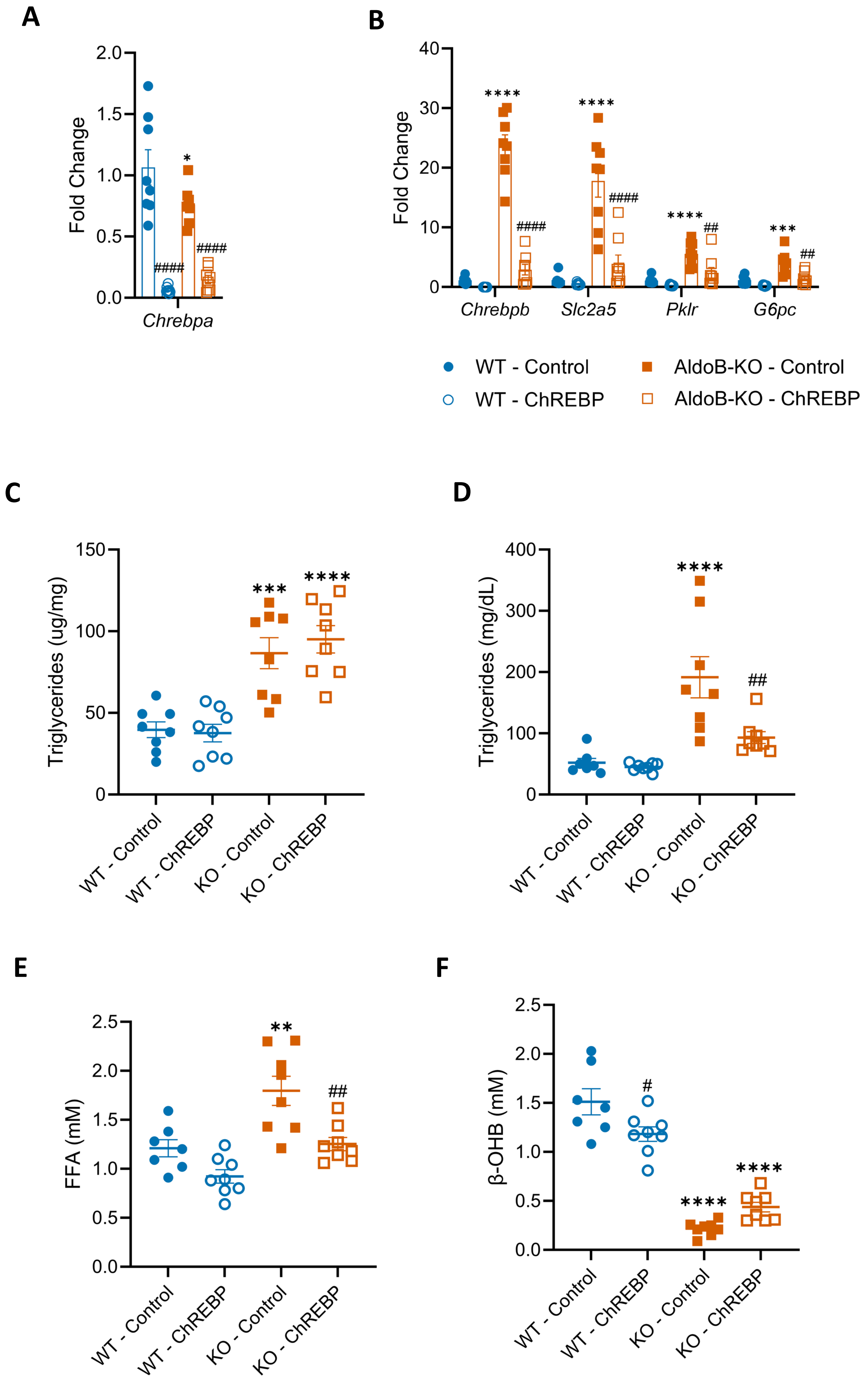

Supplemental Figure 6

A

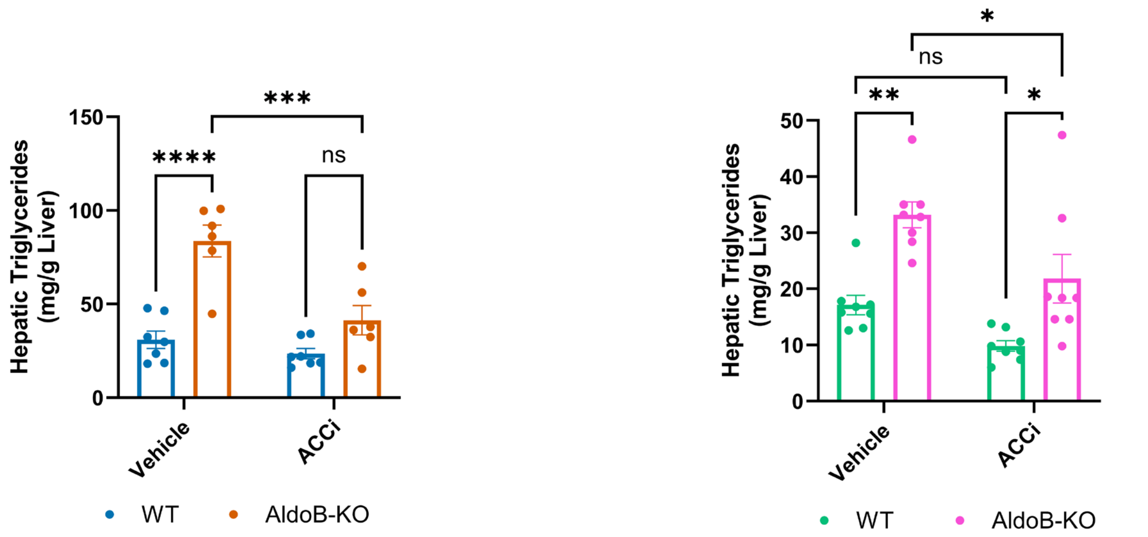


B

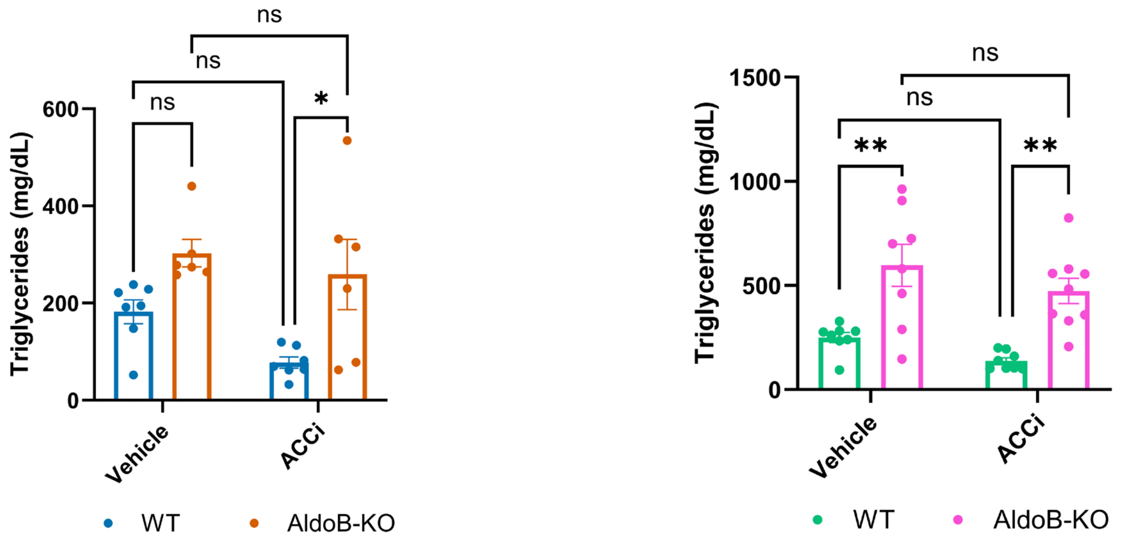


C

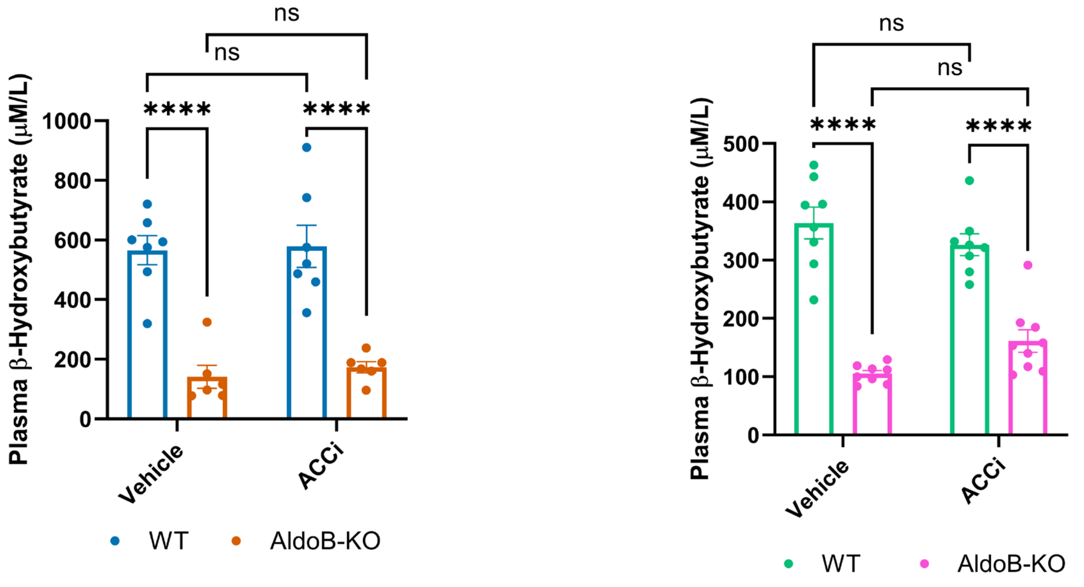

# Supplemental Figure 7

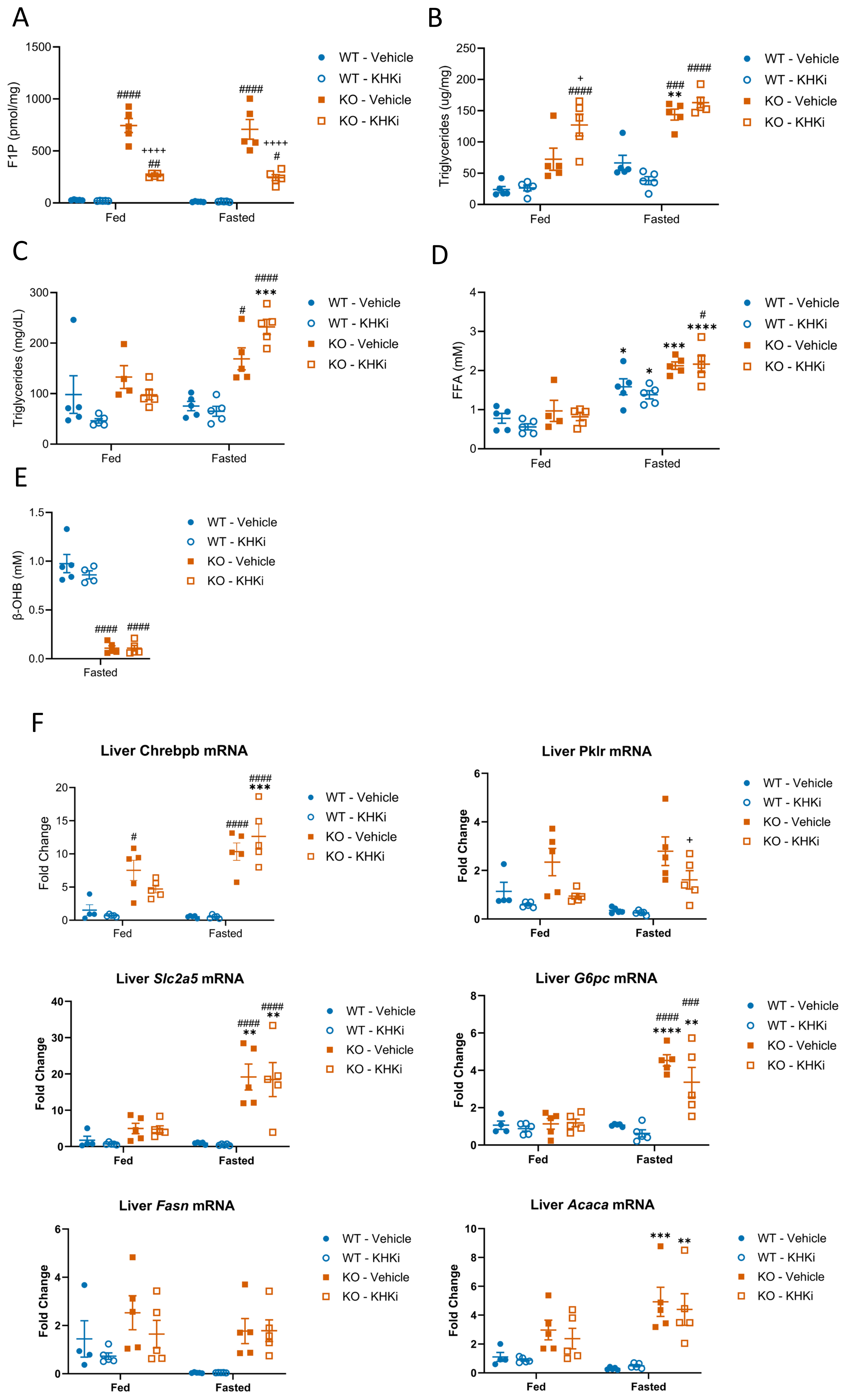

# Supplemental Table 1

| Kidney H&E | HOM Female | | | | | | | | WT Female | | | | | | | | | | | HOM Male | | | | | | | | | | | | WT Male | | | | | | | | |
|---|---|---|---|---|---|---|---|---|---|---|---|---|---|---|---|---|---|---|---|---|---|---|---|---|---|---|---|---|---|---|---|---|---|---|---|---|---|---|---|---|
| Diet | D16121902 | | | | 2920X | | | | D16121902 | | | | | 2920X | | | | | | D16121902 | | | | | | 2920X | | | | | | D16121902 | | | | 2920X | | | | |
| Animal ID | 49 | 51 | 53 | 56 | 50 | 52 | 54 | 55 | 57 | 59 | 60 | 63 | 64 | 58 | 61 | 62 | 65 | 66 | 67 | 70 | 72 | 73 | 75 | 77 | 79 | 68 | 69 | 71 | 74 | 76 | 78 | 81 | 83 | 85 | 86 | 80 | 82 | 84 | 87 | 88 |
| Tubular basophilia | 0 | 0 | 0 | 0 | 0 | 0 | 0 | 0 | 0 | 0 | 0 | 0 | 0 | 0 | 0 | 1 | 0 | 0 | 0 | 0 | 0 | 0 | 1 | 1 | 0 | 0 | 0 | 1 | 1 | 1 | 0 | 1 | 0 | 0 | 0 | 1 | 0 | 0 | 0 | 1 |
| Interstitial mononuclear cell infiltrate | 0 | 0 | 0 | 0 | 0 | 0 | 0 | 0 | 0 | 0 | 0 | 0 | 0 | 0 | 1 | 1 | 0 | 0 | 0 | 0 | 0 | 0 | 1 | 1 | 0 | 2 | 0 | 0 | 0 | 0 | 0 | 0 | 0 | 0 | 0 | 0 | 0 | 0 | 0 | 0 |
| Thickened tubular/glomerular basement membrane | 0 | 0 | 0 | 0 | 1 | 0 | 0 | 0 | 0 | 0 | 0 | 0 | 0 | 0 | 0 | 0 | 0 | 0 | 0 | 0 | 0 | 0 | 1 | 1 | 0 | 0 | 0 | 0 | 0 | 1 | 0 | 0 | 0 | 0 | 0 | 0 | 0 | 1 | 0 | 1 |
| Protein cast and/or tubular dilation | 0 | 0 | 0 | 0 | 0 | 0 | 0 | 0 | 0 | 0 | 0 | 0 | 0 | 0 | 0 | 0 | 1 | 0 | 0 | 0 | 1 | 0 | 0 | 1 | 0 | 0 | 0 | 0 | 1 | 1 | 1 | 1 | 1 | 0 | 0 | 1 | 0 | 1 | 0 | 1 |
| dilation: pelvis | 0 | 0 | 0 | 0 | 2 | 0 | 1 | 0 | 0 | 2 | 0 | 4 | 0 | 4 | 0 | 0 | 0 | 0 | 0 | 0 | 0 | 0 | 0 | 0 | 0 | 1 | 0 | 0 | 0 | 0 | 0 | 0 | 0 | 0 | 0 | 0 | 0 | 0 | 0 | 0 |
| Brown pigment: tubular epithelium, corticomedullary junction | 2 | 0 | 0 | 0 | 0 | 0 | 0 | 0 | 0 | 0 | 0 | 0 | 0 | 0 | 0 | 0 | 0 | 0 | 0 | 0 | 0 | 0 | 0 | 0 | 0 | 0 | 0 | 0 | 0 | 0 | 0 | 0 | 0 | 0 | 0 | 0 | 0 | 0 | 0 | 0 |
| Tubular hypertrophy/dilation/vacuolation, corticomedullary junction | 2 | 1 | 1 | 1 | 1 | 2 | 2 | 2 | 0 | 0 | 0 | 0 | 0 | 0 | 0 | 0 | 0 | 0 | 0 | 0 | 0 | 0 | 0 | 0 | 0 | 0 | 0 | 0 | 0 | 0 | 0 | 0 | 0 | 0 | 0 | 0 | 0 | 0 | 0 | 0 |

Kidney: Tubular hypertrophy/dilation/vacuolation with some intraluminar proteinaceous material, mainly at the corticomedullary junction was present in all HOM females with both diets. In this strain/study, severity was higher in the 2920X diet. All other renal changes seen are associated with early sign of chronic progressive nephropathy (characterized by tubular basophilia, protein cast and/or tubular dilation, thickened tubular/glomerular basement membrane and interstitial mononuclear infiltrates) which is a degenerative lesion of the kidney commonly encountered in rodents and more prominent in males. This change is not considered to be related to the AldoB null phenotype.

# Supplemental Table 2

## Changes in hsa03320 PPAR signaling pathway

| | symbol | entrez_id | logFC |
|---|---|---|---|
| 1 | ACADM | 34 | -1.31 |
| 2 | ACSL1 | 2180 | -1.35 |
| 3 | APOA1 | 335 | -1.45 |
| 4 | CPT1B | 1380 | -2.22 |
| 5 | CYP7A1 | 1580 | -1.46 |
| 6 | EHHADH | 1960 | -2.98 |
| 7 | FABP1 | 2170 | -2.52 |
| 8 | FABP7 | 2170 | -3.4 |
| 9 | FADS2 | 9420 | -2.68 |
| 10 | ME1 | 4200 | 2.79 |
| 11 | PLIN2 | 123 | 1.2 |
| 12 | PLIN5 | 441000 | -1 |
| 13 | SCD | 6320 | 4.98 |
| 14 | SLC27A5 (FATP5) | 11000 | -1.5 |
| 15 | SORBS1 | 10600 | -1.06 |

# Supplemental Table 3

## Changes in hsa04976 Bile secretion pathway

| | symbol | entrez_id | logFC |
|---|---|---|---|
| 1 | ABCG2 | 9430 | -1.72 |
| 2 | ABCG5 | 64200 | -5.06 |
| 3 | ABCG8 | 64200 | -6.35 |
| 4 | ATP1B1 | 481 | 1.77 |
| 5 | ATP1B2 | 482 | 1.6 |
| 6 | BAAT | 570 | -1.38 |
| 7 | CYP7A1 | 1580 | -1.46 |
| 8 | NCEH1 | 57600 | -1.68 |
| 9 | NR0B2 (SHP) | 8430 | -3.38 |
| 10 | SCARB1 | 949 | -1.17 |
| 11 | SLC2A1 | 6510 | -1.77 |
| 12 | SLC10A1 | 6550 | -1.04 |
| 13 | SLC22A7 | 10900 | -1.54 |
| 14 | SLC27A5 | 11000 | -1.5 |
| 15 | SULT2A1 | 6820 | -3.95 |

# Supplemental Table 4

*Significantly Altered Metabolites in AldoB KO vs WT rats in Fed State*

| Metabolite | FC | log2(FC) | raw.pval | -LOG10(p) |
|---|---|---|---|---|
| Fructose 1-phosphate | 318.33 | 8.3144 | 8.98E-14 | 13.047 |
| Fructose 1,6-diphosphate | 29.438 | 4.8796 | 2.17E-09 | 8.6635 |
| Glucose 1-phosphate | 2.5592 | 1.3557 | 3.90E-05 | 4.4087 |
| Sarcosine | 1.9235 | 0.94373 | 0.00062788 | 3.2021 |
| Erythrose 4-phosphate | 105.04 | 6.7148 | 0.0018093 | 2.7425 |
| Dihydroxyacetone phosphate | 2.4898 | 1.316 | 0.0035254 | 2.4528 |
| Pro | 2.5436 | 1.3469 | 0.0054961 | 2.2599 |
| Cystathionine | 1.977 | 0.98329 | 0.0076808 | 2.1146 |
| Ala | 1.5833 | 0.66295 | 0.01226 | 1.9115 |
| Fructose 6-phosphate | 2.161 | 1.1117 | 0.018483 | 1.7332 |

# Supplemental Table 5

*Significantly Altered Metabolites in AldoB KO vs WT rats in Fasted State*

| **Metabolite** | **FC** | **log2(FC)** | **raw.pval** | **-LOG10(p)** |
|---|---|---|---|---|
| Fructose 1-phosphate | 6.88 | 2.7824 | 1.11E-12 | 11.953 |
| Pyruvic acid | 4.3232 | 2.1121 | 1.37E-07 | 6.8624 |
| Glycerol 3-phosphate | 4.3601 | 2.1244 | 3.64E-07 | 6.4389 |
| 2-Oxoglutaric acid | 60.189 | 5.9114 | 4.23E-07 | 6.3742 |
| Acetyl CoA | 0.30342 | -1.7206 | 5.88E-07 | 6.2308 |
| Lactic acid | 2.2016 | 1.1386 | 1.03E-06 | 5.9856 |
| CoA | 0.39289 | -1.3478 | 1.14E-06 | 5.9445 |
| ADP | 0.59243 | -0.75528 | 1.05E-05 | 4.9805 |
| Guanosine | 0.43317 | -1.207 | 1.25E-05 | 4.9029 |
| Fumaric acid | 0.34885 | -1.5193 | 1.61E-05 | 4.7935 |
| 2-Hydroxyglutaric acid | 2.0808 | 1.0571 | 1.76E-05 | 4.7536 |
| Malic acid | 0.34816 | -1.5222 | 1.81E-05 | 4.7414 |
| 2-Phosphoglyceric acid | 15.203 | 3.9262 | 2.57E-05 | 4.5904 |
| Phosphoenolpyruvic acid | 23.448 | 4.5514 | 2.76E-05 | 4.5598 |
| 3-Phosphoglyceric acid | 15.986 | 3.9988 | 4.16E-05 | 4.3811 |
| Adenosine | 0.26256 | -1.9293 | 4.91E-05 | 4.309 |
| AMP | 0.38369 | -1.382 | 9.12E-05 | 4.0402 |
| PRPP | 9.9065 | 3.3084 | 0.00010218 | 3.9906 |
| GDP | 0.62443 | -0.67938 | 0.00015706 | 3.8039 |
| Hypoxanthine | 0.10193 | -3.2944 | 0.00019428 | 3.7116 |
| 2-Oxoisovaleric acid | 1.8943 | 0.92165 | 0.00027994 | 3.5529 |
| Ala | 2.0758 | 1.0537 | 0.00028414 | 3.5465 |
| Inosine | 0.20982 | -2.2528 | 0.00037701 | 3.4237 |
| Adenylosuccinic acid | 0.27717 | -1.8511 | 0.00094997 | 3.0223 |
| Xylulose 5-phosphate | 9.1566 | 3.1948 | 0.0012847 | 2.8912 |
| Asp | 0.45231 | -1.1446 | 0.0015672 | 2.8049 |
| Xanthine | 0.23589 | -2.0838 | 0.0025587 | 2.592 |
| Guanine | 0.18724 | -2.417 | 0.0030904 | 2.51 |
| N,N-Dimethylglycine | 0.6556 | -0.60911 | 0.0042132 | 2.3754 |
| N-Carbamoylaspartic acid | 1.7358 | 0.79564 | 0.0046832 | 2.3295 |
| Î²-Ala | 0.58504 | -0.77339 | 0.0050265 | 2.2987 |
| Dihydroxyacetone phosphate | 6.5118 | 2.7031 | 0.0071311 | 2.1468 |
| Cys | 0.33442 | -1.5803 | 0.013742 | 1.8619 |
| Ser | 0.60859 | -0.71647 | 0.017802 | 1.7495 |
| Fructose 1,6-diphosphate | 2.1598 | 1.1109 | 0.039548 | 1.4029 |
